# Altercentric Memory Error at 9 Months But Correct Object Memory by 18 Months Revealed in Infants’ Pupil

**DOI:** 10.1111/desc.70016

**Published:** 2025-03-30

**Authors:** Anna‐Lena Tebbe, Katrin Rothmaler, Hannah Elena Zielke, Robert Hepach, Charlotte Grosse Wiesmann

**Affiliations:** ^1^ Research Group ‘Milestones of Early Cognitive Development’ Max Planck Institute for Human Cognitive and Brain Sciences Leipzig Germany; ^2^ University of Leipzig Leipzig Germany; ^3^ Department of Experimental Psychology University of Oxford Oxford UK; ^4^ Cognitive Neuroscience Lab Department of Liberal Arts and Sciences University of Technology Nuremberg Nuremberg Germany

**Keywords:** action prediction, altercentric bias, infancy, memory errors, pupillometry, theory of mind

## Abstract

It was recently proposed that infants have a memory bias for events witnessed together with others. This may allow infants to prioritize relevant information and to predict others' actions, despite limited processing capacities. However, when events occur in the absence of others, for example, an object changes location, this would create altercentric memory errors where infants misremember the object's location where others last saw it. Pupillometry presents a powerful tool to examine the temporal dynamics of such memory biases as they unfold. Here, we showed infants aged 9 (*N *= 97) and 18 months (*N *= 79) videos of an agent watching an object move to one of two hiding locations. The object then moved from location A to B, which the agent either missed (leading to her false belief) or witnessed (true belief). The object subsequently reappeared either at its actual or, surprisingly, its initial location. As predicted by the altercentric theory, 9‐month‐old infants expected the object where the agent falsely believed it to be and not where it really was, as indicated in their pupil dilation. In contrast, 18‐month‐old infants seemed to remember the object's actual location. Infants’ memory errors did not predict correct action anticipation when the agent reached into one of the locations to retrieve the object. This indicates that infants show altercentric memory errors at a young age, which vanish in the second year of life. We suggest that this bias helps young infants to learn from others, but recedes as they become more capable of acting on the world themselves.

## 1 Introduction

Young infants are strongly dependent on others. Not only do they rely on caregivers given their own limited motoric abilities, but early development also involves continuous learning processes that are strongly guided by others (Hunnius and Bekkering 2010, 2014; Southgate 2020). As a result, understanding the targets of others’ attention and the objects they refer to when communicating is vital for infants’ learning. This provides them with an adaptive way of navigating their environment (Woodward 2009; Yoon et al. 2008). Thus, infants naturally pay close attention to the actions and attentional cues of others (Brooks and Meltzoff 2002; Meltzoff and Brooks 2017).

**Summary**

- Pupil data suggests that 9‐month‐old infants show altercentric memory errors when another person misses an object's change of location leading to their false belief.
- Eighteen‐month‐old infants, in contrast, seem to remember the object's location correctly, as indicated by increased pupil dilation to reality incongruent outcomes.
- This memory bias was not related to correct action prediction, but may serve as an important early learning function.

Indeed, newborns already show a preference for faces over non‐social stimuli (Johnson et al. 1991; Valenza et al. 1996), rudimentary forms of gaze following (Farroni et al. 2002, Farroni et al. 2004), and young infants are thought to excel at predicting others’ actions (Baillargeon 2004; Luo and Baillargeon 2005; Song and Baillargeon 2007; Woodward 1998, but see Ganglmayer et al. 2019; Sirois et al. 2023). The attention of others gains particular importance toward the end of the first year of life, from around 8 to 10 months, when infants begin to engage in joint attention with others toward targets in their environment (Striano and Bertin 2005; Tomasello 1995). In this period, infants’ memory is enhanced when their attention to events is socially cued by the actions, gaze, or communication of others (Howard and Woodward 2019; Michel et al. 2019; Thiele et al. 2021; Yoon et al. 2008). Further, infants’ expectations of finding an object in a certain location were enhanced when another agent had seen the object there last (Kampis and Kovács 2021; Kovács et al. 2010).

Recently, it has been proposed that infants may even prioritize what others have seen over their own view (Grosse Wiesmann and Southgate 2021; Southgate 2020). Specifically, the altercentric account suggests that infants remember events better that they have seen together with others than events later witnessed alone (Grosse Wiesmann and Southgate 2021; Southgate 2020). In case changes occur in the absence of another agent, this would lead to altercentric memory errors where infants misremember situations as seen by others and fail to update their representation of the situation after the change has occurred. Although leading to memory errors in some situations, this bias would allow infants to encode the world from the perspectives of others and thus follow the referents of their attention and communication. Moreover, it was proposed that this memory bias would allow infants to predict others’ actions in situations where the others’ perspective should differ from their own (Grosse Wiesmann and Southgate 2021; Southgate 2020).

Indeed, traditionally, reasoning about diverging perspectives has been assumed to be cognitively effortful and not to emerge before 4 years (e.g., Perner 1991; Rakoczy 2022). Yet, a growing body of research suggests that preverbal infants already correctly predict how an agent with a different perspective will act (e.g., Rakoczy 2022; Scott and Baillargeon 2017). These findings raise the question of how young infants would manage diverging perspectives, despite their limited processing capacities. The altercentric theory resolves this puzzle by proposing that infants do not, in fact, represent several diverging perspectives, but predict the others’ actions based on their own biased memory of events (Southgate 2020). Thus, when another agent saw an object in one location but does not see that it has been moved to a different location in the meantime, infants would misremember this object in the first location in line with the agent's belief, allowing them to predict where the agent will search for the object (e.g., Grosse Wiesmann and Southgate 2021).

A recent study found the first indications of such an altercentric bias in the first year of life (Manea et al. 2023). Specifically, 8‐month‐old infants seemed to misremember an object's location if an agent had only seen it in a first location but did not witness its transfer to a second location, as indicated by longer looking times to reveal the first compared to the second location as empty. However, infants did not show memory of the object's location when the agent had witnessed the transfer, calling for a replication of the observed altercentric bias with a different paradigm. Further, the altercentric bias was not observed at 12 months where infants showed equal looking times for both outcomes. This raises the question of whether altercentric biases may only be observed early in infancy.

Indeed, it has been suggested that the altercentric bias may decrease in the second year of life as infants develop a self‐concept proposed to highlight the relevance of their own perspective compared to that of others (Grosse Wiesmann and Southgate 2021; Southgate 2020). The period from around 18 months is characterized by important developments of the self‐concept (e.g., Kampis et al. 2022; Moore 2007; Rochat 2010), as indicated, amongst others, by emerging mirror self‐recognition (MSR) (Amsterdam 1972; Rochat 2010). Further, as infants become more capable of acting on the world, an accurate representation of the environment gains increasing importance over the view of others. In line with this, a recent study found that infants show a shift from better memory for other‐relevant to self‐relevant content with MSR around 18 months (Grosse Wiesmann et al. 2024). These arguments and findings motivate the hypothesis that altercentric memory errors may disappear by 18 months of age, possibly in relation to MSR.

Against this background, in the present study, we addressed the following questions: (1) Can the previously observed altercentric memory error (Manea et al. 2023) be replicated with a different, potentially more sensitive measure? (2) How does this memory bias develop in the second year of life (particularly, at 18 months as MSR emerges)? (3) Can infants’ altercentric memory errors predict their correct expectations of how an agent with a different perspective (i.e., a false belief about an object) will act?

To test this, we presented 9‐ and 18‐month‐old infants with videos of an agent watching an object moving into one of two locations. The agent then either witnessed how the object moved from location A to B (leading to her true belief, TB) or missed this transfer (leading to her false belief, FB). The object then reappeared either at its actual location (reality congruent) or from its initial location (reality incongruent). We predicted that, if infants show an altercentric memory error, they should expect the object in its initial location, namely, where the agent last saw it but not where it actually was. In contrast, if 18‐month‐old infants remembered the correct object location, they should expect the object in its second, actual location, irrespective of the agent's belief.

While the study by Manea et al. (2023) had used infants’ cumulative looking times as a measure of their expectations, we measured infants’ pupil size. In contrast to looking times or gaze, pupillary responses are functionally related to increased attention and cognitive effort (Hepach and Westermann 2016; Sirois and Jackson 2012). Further, looking time in contrast to pupillometry cannot track information processing dynamics over time (e.g., Haith 1998; Sirois and Jackson 2007), but only shows which stimuli infants are able to discriminate without providing insight about underlying processes. Pupil size is therefore considered a particularly reliable and sensitive measure of infants’ surprise (Dörrenberg et al. 2018; Hepach 2024; Jackson and Sirois 2022; Mayer and Liszkowski 2025; Pätzold and Liszkowski 2020) and cognitive effort (Kaldy and Blaser 2020).

To test whether altercentric memory errors would be related to infants' correct predictions of the agent's actions, we added a second block, in which we presented infants with analogous videos where, instead of revealing the object at the end of the trial, the agent reached into one of the two locations to retrieve it. Thus, one block targeted infants’ object memory, and the other block probed its relation to infants' correct action prediction. Further, we predicted that infants who showed altercentric memory errors (i.e., remembering the objects’ location where the agent believes it to be) would also show better action prediction in the false belief trials of the other block. In addition to the preregistered analyses, we measured infants’ joint attention, gaze‐following abilities, and MSR in a subset of infants who had completed these tasks in the context of another behavioral study. We predicted infants’ joint attention to be positively related with showing altercentric biases at 9 months. Further, at 18 months, infants’ MSR was predicted to be negatively related with their altercentric memory errors (Grosse Wiesmann and Southgate 2021; Southgate 2020; Yeung et al. 2022).

## 2 Methods

The study was preregistered at https://aspredicted.org/5RY_616. Data and analysis scripts are provided on OSF (https://osf.io/rbktq/?view_only=5e98911ddaec4f0b8ac8e45261711e59).

### 2.1 Participants

The study reports data from *N *= 176 typically developing, full‐term born infant participants (*N *= 97 infants aged 8–10 months, 42 female; *M_age_ *= 9.4 months, *SD *= 25.37 days; and *N *= 79 infants aged 17–19‐month‐old, 41 female; *M_age_ *= 18.6 months, *SD *= 19.89 days). We did not record detailed information on participants' socioeconomic or ethnic backgrounds. Our database is largely composed of families from a predominantly white demographic with mid to high socioeconomic status. Families were recruited in an urban Western, industrialized context in Germany. Twenty‐eight additional infants were tested but had to be excluded from further analyses due to technical problems (*N *= 18), experimenter error (*N *= 2), or not contributing sufficient trials (*N *= 8). We recorded data following a Bayesian Sequential Testing scheme (Mani et al. 2021; Schönbrodt et al. 2017), details see below and Figure S1 in Supporting Information S1. The analyses converged at *N *= 58 for the 9‐month‐old infants and *N *= 29 for the 18‐month‐old infants, but because of the complex nature of the preprocessing and analysis streams, we continued testing and reported the full sample here. The results at the stopping criterium are similar (see Supporting Information S1). We obtained written informed consent from the parents before participation, parents received 10€ for their travel expenses, and infants a little gift for their participation. The study received approval from the ethics committee of the University of Leipzig.

### 2.2 Eye‐Tracking Stimuli and Procedure

Infants gaze and pupil size da ta were recorded using a Tobii X120 eye tracker (Tobii Technology, Stockholm, Sweden) with 120 samples per second (i.e., 120 Hz). The eye‐tracker was positioned approximately 60 cm away from the participant with an angle of 30°. Caregivers wore sunglasses during the recording and were asked not to intervene during testing. Animated videos were created using the graphics software Blender (v. 2.8, www.blender.org) and presented full screen on a screen of 1280 × 1024 pixels with Tobii Studio (v. 2.2.8). Lighting conditions were assessed using a luxmeter and were kept the same for all participants (∼16–18 Lux; lux meter angled toward the wall behind the screen with a distance of approx. 60 cm). We created a testing cubicle with room‐divide‐boards. Before beginning the experimental session, each infant passed a five‐point infant calibration implemented in Tobii Studio.

Infants were presented with two different blocks of videos: one block tested infants’ object location memory (Object Memory), the other block their capacity to predict where an agent would search for the object (Action Prediction). In each block, infants first watched two familiarization trials which were followed by four test trials. The test trials differed with respect to the agent's belief (true belief vs. false belief) and the outcome that was shown (reality congruent vs. reality incongruent). The block order was counterbalanced across participants.

In the familiarization trials, the agent waved and smiled at the infant. An object appeared from the top center of the screen and fell straight down on the ground (while an engaging sound was played) in the middle between two hollow locations. The object then moved into location A. Once the object was fully hidden, the agent redirected her gaze to the center bottom of the screen and briefly paused. In the familiarization trials of the Object Memory block, the agent then observed how the object was revealed from its location. In the Action Prediction block, instead of revealing the object, the agent reached the location with the object. The familiarization trials were intended to familiarize infants with the scene and its expected outcome, that is, that the object would reappear at the end of the trial in the Object Memory block, and that the agent would reach toward the object in the Action Prediction block. In each of the two blocks, the object entered the left location in one familiarization trial and the right location in the other familiarization trial. The order of the sides was counterbalanced across participants.

Test trials differed from the familiarization trials in that, after entering its first location (location A), the object rolled into the other location (location B). In False Belief^1^ test trials, a curtain was lowered so that the agent was occluded and could not see how the object moved from A to B. Once the object was fully occluded in location B, the curtain was raised again.

In True Belief trials, the agent followed this change with her gaze, and the curtain was only lowered and raised again, once the object had fully disappeared in location B. Note that in both True and False Belief trials the curtain was raised at the same time (13.4–15 s). This was followed by an anticipation phase (15–17 s), in which a brief chime was played. This period showed an identical scene for TB and FB conditions. There were no differences in the baseline period between outcomes.

**FIGURE 1:**
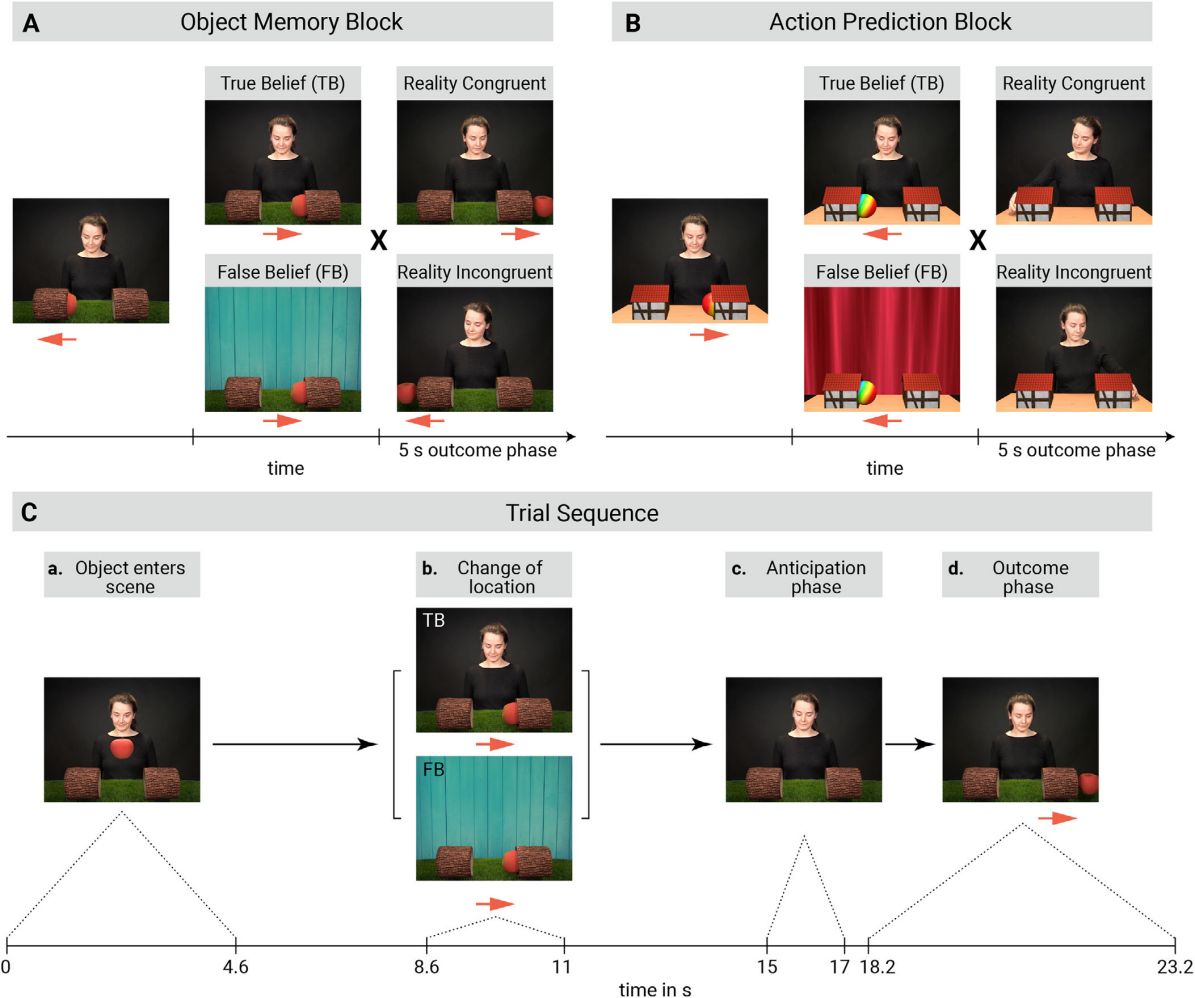
Schematic illustration showing the design of the two experimental blocks (A) Object Memory block; (B) Action Prediction block. In test trials of both blocks, an object appeared in the scene and moved into one of two possible locations. The subsequent change of its location was either observed by the agent (True Belief) or missed (False Belief). After an anticipation phase, a reality congruent or incongruent outcome was presented. In the Object Memory block (A), the object reappeared from the reality congruent or incongruent location. In the Action Prediction block (B), the agent searched for the object in the reality congruent or incongruent location. (C) Schematic illustration showing the time course and sequence of events in an exemplary Object Memory test trial with a 2 s anticipation phase (c) and a 5 s outcome time window (d). The timing was identical for the Action Prediction block.

Finally, in the outcome scene, the reality‐congruent or ‐incongruent outcome was presented. In the Object Memory block, the object reappeared from its actual or initial location. In the Action Prediction block, the agent reached for the actual object location or the incongruent side. For a schematic illustration of the scene and time course see Figure 1. The timing of the trials in the two blocks was identical, with a total duration of 23.2 s per test trial. Each trial was preceded by an attention‐getter, which showed a shaking colorful object displayed at the center of the screen accompanied by a short engaging sound. After the attention‐getter, a black screen was presented for 1 s before the actual test trial began.

The order of the test trials concerning Belief (True vs. False Belief) and Outcome (congruent vs. incongruent) was pseudorandomized across participants but kept the same in both blocks within the same participant. This was done to exclude that order effects would explain differences between the two tasks. The sides of the reality congruent outcome and the presented outcome were counterbalanced (see Supporting Information S2). There was no effect of first trial congruency on the pupil dilation response (see Supporting Information S3) and no effect of block order on the Object Memory block. In the Action Prediction block, there was an effect of block order on the false belief trials (see Supporting Information S3).

### 2.3 Additional Measures

#### 2.3.1 Joint Attention

In addition, we acquired the Dimensional Joint Attention task (DJAA; Elison et al. 2013), testing infants' capacity to respond to attention‐sharing signals from the experimenter. Each infant was exposed to up to four possible joint attention situations (trials). For each trial, the target object was one of ten novel objects spread within the testing room, approximately 1–2 m away from the infant and located in a position that required a gaze shift of approximately 90°. Each trial was composed of four levels increasing in cue redundancy, and the trial was stopped as soon as the infant responded to the cues, determining the infant's level (procedure described in Elison et al. 2013 and Stallworthy et al. 2022). The higher the level (i.e., the higher the redundancy until the infant responded to the cues), the easier the task, and thus, the lower the infants’ score. The score ranged from 4 points (for level 1) to 1 point (for level 4). Infants’ total score was computed as the average score of all four trials.

#### 2.3.2 Mirror Self Recognition

As a measure of developing self‐awareness, we utilized the MSR test and classified infants into recognizers and non‐recognizers (Amsterdam 1972), following the protocol by Kampis et al. (2022) and Grosse Wiesmann et al. (2024). Children were classified as mirror passers if they touched a mark that had surreptitiously been placed on their faces or produced verbal self‐reference (i.e., a first‐person pronoun or their own name) when seeing themselves in the mirror, either spontaneously or when prompted. Children received a score of 1 if they passed the mirror test, and a score of 0 if they did not pass.

### 2.4 Preprocessing of Eye‐Tracking Data

For the pupil response, we analyzed the baseline corrected pupil time course and pupil dilation average in percentage change for each infant and trial in the last 5 s (i.e., the outcome phase) of each video. Preprocessing steps followed previous procedures (Hepach et al. 2012, Hepach et al. 2016) For each sample point, data were recorded for both the left and the right eye. First, we removed unrealistic values (i.e., pupil sizes < 0 or fixations outside the screen). For each eye, data were filtered with a percentile cutoff filter to minimize the effects of extreme sample‐to‐sample differences. This filter excluded samples which, in their averaged absolute differences from the previous and subsequent samples, exceeded the 90th percentile of the respective distribution (see Hepach et al. 2016). In the next step, we linearly interpolated missing samples if the gap between two samples was not greater than eight samples (i.e., ∼70 ms with a sampling frequency of 120 Hz). Data for both eyes were then averaged, filtered, and interpolated again by applying the same filter and interpolation settings as before. For Baseline correction, we calculated the average pupil dilation during a 500 ms time window within the anticipation phase (i.e., from 16.5 to 17 s), which directly preceded the first frame revealing the subsequent outcome location, see Figure 1. This period showed an identical scene for all conditions. There were no differences in the baseline period between outcomes. We subtracted the baseline from all subsequent values and divided the result by the baseline and segmented the dataset to the outcome phase only (5 s), resulting in 600 potential sample points per participant and trial. Individual sample points that were more than three median absolute deviations from the median were excluded from analyses (Prunty et al. 2022). Trials with less than 10% of samples within the outcome window were excluded from the pupil dilation analyses. Highly similar results were obtained when excluding trials with less than 50% of samples (see Supporting Information S4). As the choices made during the preprocessing stage (e.g., filter or baseline correction) can significantly influence the presence and extent of effects (Calignano et al. 2024; Sirois et al. 2023), we have analyzed our data with different preprocessing parameters, see Supporting Information S5 for a robustness check.

Trials in which the eye tracker recorded missing data during the object's change of location were manually coded and included if the infant looked at the screen for a minimum of 50% of the change of location time window (also see Supporting Information S6 for a control analysis of gaze samples recorded). An independent, second rater coded 25% of the respective videos. The interrater agreement between the two coders was very good (*κ *= 0.81). Infants were included if they contributed at least one trial per condition relevant to the analyses (see Supporting Information S7 for the number of included infants per analyses and Supporting Information S8 for a visualization of missing data points across trials).

For anticipatory‐looking behavior and first gaze analysis, we extracted coordinates of mapped fixations in a 2000 ms time window from 15 to 17 s after onset. This time window showed a still frame of the agent and was identical for the True and False Belief trails, see Figure 1. Fixations were defined using the Tobii fixation filter (velocity threshold of 35/s; distance threshold of 35, see Supporting Information S9 for mapped fixations during the anticipation and outcome phase. Trials in which parents interfered during trials were excluded from further analysis.

### 2.5 Statistical analyses

Statistical analyses were carried out in R (R Core Team, 2017; version 4.2.1), Matlab (R2017a; Mathworks, Inc., Natick, MA, USA) and JASP (0.17.3). We used Bayesian statistics to quantify statistical evidence for the observed effects via the Bayes Factor BF_10_ that sets the evidence for the alternative model in relation to the evidence for the null model using the bayesfactor toolbox (Krekelberg 2024) Following the classification scheme in Lee and Wagenmakers (2014) we classified a BF_10_ between 3 and 10 as moderate evidence, between 10 and 30 as strong, and between 30 and 100 as very strong evidence *for* an effect. In turn, a BF_10_ between 1/3 and 1/10 was classified as moderate evidence, between 1/10 and 1/30 as strong, and between 1/30 and 1/100 as very strong evidence *against* an effect. As no prior information on the expected data distribution in infant pupillometry change of location tasks was available, we chose default priors for all our model comparisons (Rouder et al. 2012). Although, in the case of our study, there was insufficient previous knowledge about the distribution of infant pupil data to choose informed priors, it is important to keep in mind that by choosing default priors, the probability of statistical null‐findings may have been inflated (Smid and Winter 2020; Williams et al. 2017). In case of major deviations from normality as indicated by q‐q‐plots (see Figs. S3 and S4 in Supporting Information S10), we used a Wilcoxon signed rank test as a non‐parametric alternative to the preregistered *t*‐tests.

### 2.6 Sampling approach

We determined our sample size following a Bayesian sequential testing approach (Mani et al. 2021; Schönbrodt et al. 2017). That is, data collection was continued until the Bayes Factor (BF) exceeded a predetermined threshold of BF = 3 or BF = 1/3 corresponding to moderate evidence for or against our main hypothesis (i.e., the Bayesian LMMs comparing the reality congruent and incongruent outcome in false belief trials for each age group). We tested a minimum sample of *N *= 50 healthy full‐term born infants (25 infants per age group) to control for false negatives and positives (Schönbrodt and Wagenmakers 2018).

## 3 Results

### 3.1 Object Memory Block

#### 3.1.1 Object Memory—Pupil Dilation

To assess whether the pupil dilation response within the Object Memory block was modulated by the expectation of the infant and the belief of the agent, we computed Bayesian Linear Mixed Models (LMM) contrasting the different outcomes in False Belief and True Belief trials for both age groups separately with the relative change in pupil dilation as dependent variable, outcome as fixed variable and subject as random effect.

##### 3.1.1.1 Nine‐Month‐Old Infants

**FIGURE 2:**
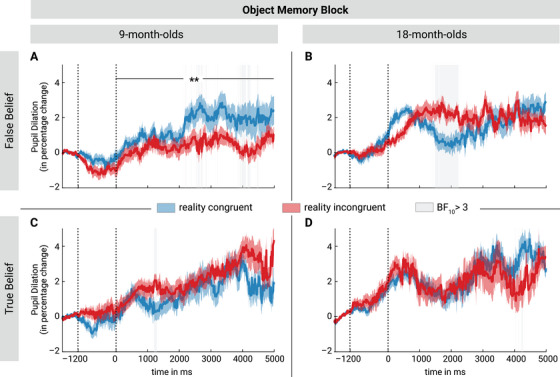
Baseline‐corrected percentage change in pupil dilation during the Object Memory block for the two age groups in False Belief trials (panels A and B) and in True Belief trials (panels C and D). The pupil dilation was baseline‐corrected with a 500 ms segment from −1700 to −1200 ms relative to the outcome phase onset. The object reappeared from its location in the time window from −1200 to 0 ms, followed by a 5000 ms time window in which the pupil response was analyzed (outcome phase). (A) 9‐month‐old infants showed larger pupil dilation to reality congruent outcomes (blue) compared to incongruent outcomes (red). (B) Conversely, at 18 months, infants exhibited the opposite pattern, showing larger pupil dilation in response to incongruent outcomes in the False Belief trials in a time window from approx. 1500 to 2240 ms. This was not the case in the True Belief control condition (C and D). Red and blue shading represents the standard error (SE), the black line indicates an average difference over the entire outcome phase (** indicating strong evidence, BF_10_ = 15.74), and the grey shading indicates BF_10_ values above 3 in the dynamic analysis.

We computed Bayesian LMMs contrasting the two outcomes at each time point starting from 1000 ms post‐onset. This showed moderate evidence for greater pupil dilation for the reality‐congruent than the reality‐incongruent outcome in two‐time windows after the object started appearing (BF_10_ repeatedly exceeded our predefined threshold ranging between 3 and 44.18 in the time windows 2216 ms—2875 ms and 3883 ms—4258 ms, see Figure 2). Similarly, when averaging across the entire 5 s outcome time window, 9‐month‐old infants’ pupil size was larger during the reality‐congruent than the reality‐incongruent outcome in False Belief trials (BF_10_ = 15.74; congruent: *N *= 60, *M *= 0.015, *SD *= 0.031; incongruent: *N *= 56, *M *= 0.001, *SD *= 0.02). This indicates that 9‐month‐old infants were surprised to see the object revealed in its current location and expected it to appear where the agent believed it to be, as predicted by the altercentric theory. In contrast, there was evidence against such a difference in the control condition (i.e., when the agent held a True Belief). Instead, in True Belief trials, there was evidence for higher pupil dilation for the reality incongruent compared to congruent outcome in a very short time window between 1225 and 1300 ms (BF_10_ between 3 and 12.12; see Figure 2; averaged across time window: congruent: *N *= 38, *M *= 0.009, *SD *= 0.024; incongruent: *N *= 46, *M *= 0.019, *SD *= 0.026; BF_10_ = 0.33; see Supporting Information S11). Additional analyses showed moderate evidence for an interaction of Outcome and Belief, BF_10_ = 4.54, for the Object Memory block in the 9‐month‐old infants, confirming that infants showed larger pupil dilation for congruent outcomes in the False Belief but not the True Belief control condition (see Supporting Information S12). Thus, in line with the altercentric theory, 9‐month‐old infants seemed to misremember the object in its first location when the agent held a false belief, but not when the agent had witnessed the transfer (True Belief).

##### 3.1.1.2 Eighteen‐Month‐Old Infants

In contrast to the 9‐month‐old infants, the 18‐month‐old infants showed moderate to extreme evidence for a greater pupil dilation for the reality‐incongruent compared to the reality‐congruent outcome in False Belief trials (BF_10_ repeatedly exceeded our predefined threshold, ranging between 3 and 291.21 in the time window from 1508 to 2241 ms after the object started appearing). This indicates that 18‐month‐old infants were surprised about outcomes in which the object reappeared from the empty (reality incongruent) location. Thus, 18‐month‐old infants showed the opposite pattern of 9‐month‐old infants, showing surprise when the scene violated reality. When averaging the pupil dilation across the whole 5 s outcome phase, there was moderate evidence against a difference between outcomes (BF_10_ = 0.14, congruent: *N *= 55, M = 0.015, SD = 0.026; incongruent: *N *= 47, M = 0.016, SD = 0.018). As for the younger infants, there was moderate evidence against a difference in the True Belief control condition (dynamical analysis: see Figure 2, average across outcome phase: BF_10_ = 0.18, congruent: *N *= 47, M = 0.018, SD = 0.025; incongruent: *N *= 30, M = 0.022, SD = 0.025). Thus, in contrast to 9‐month‐old infants, 18‐month‐old infants did not show any altercentric memory error but remembered the object in its correct location.

#### 3.1.2 Object Memory—Anticipatory Looking

In addition to infants’ pupil size, we also analyzed their gaze before the object was revealed as an index of the anticipated outcome (see Supporting Information S13). In line with the pupil data, 18‐month‐old infants looked more to the current object location than to the empty location both in False Belief trials (Wilcoxon test for DLS > 0: BF_10_ = 107.46,*W *= 911; $\hat{R}$ = 1.01; *N *= 56; *M *= 0.41; *SD *= 0.71; binomial test for first gaze: BF_10_ = 1.82) and in True Belief trials (BF_10_ = 23.36; *W *= 780; $\hat{R}$ = 1; *N *= 49; *M *= 0.41; *SD *= 0.76; first gaze: BF_10_ = 244.02, also see Supporting Information S13). This indicates that they remembered the correct object location equally in both conditions, independently of the agent's belief. Nine‐month‐old infants did not show any systematic anticipatory looking (see Supporting Information S13), probably reflecting immature anticipatory looking responses at this age (Elsner and Adam 2021; Krogh‐Jespersen and Woodward 2018; Reznick et al. 2000).

## 4 Action Prediction Block

To assess whether infants’ pupil and gaze responses within the Action Prediction block were modulated by the outcome (i.e., whether the agent reached for the reality congruent or incongruent location) and the agent's belief, we computed Bayesian LMMs as before.

### 4.1 Action Prediction—Pupil Dilation

#### 4.1.1 Nine‐Month‐Old Infants

**FIGURE 3:**
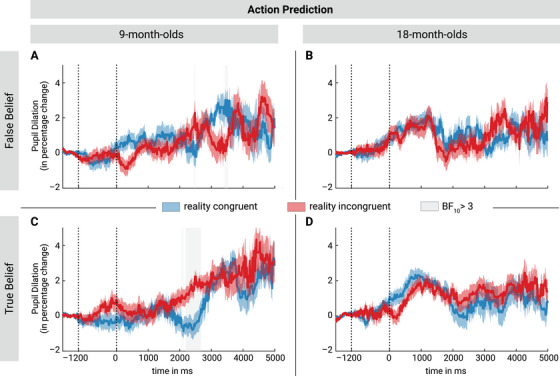
Baseline‐corrected percentage change in pupil dilation in the Action Prediction block for the two age groups in False Belief (panels A and B) and True Belief trials (panels C and D). The pupil dilation was baseline‐corrected with a 500 ms segment from −1700 to −1200 ms relative to the outcome phase onset. The agent reached for the object in the time window from −1200 ms to 0 ms, followed by a 5000 ms time window in which the pupil response was analyzed (outcome phase). Both 9‐month‐old infants (A) and 18‐month‐old infants (B) showed no systematic differences in pupil dilation depending on the outcome in False Belief trials. In True Belief trials, 9‐month‐old infants showed higher pupil dilation for reality‐incongruent than congruent outcomes in a time window from 2200 to 2690 ms (C), while there were no differences in pupil dilation depending on outcome in 18‐month‐old infants (D). Red and blue shading represents the standard error (SE) and gray shading indicates BF_10_ values above 3.

In the Action Prediction block, 9‐month‐old infants showed no systematic differences in pupil dilation depending on the outcome in False Belief trials (dynamic analysis: see Figure 3; average across outcome phase: BF_10_ = 0.27, congruent: *N *= 51, *M *= 0.009, *SD *= 0.03; incongruent: *N *= 51, *M *= 0.004, *SD = *0.026). In the True Belief control condition, in turn, infants showed higher pupil response to incongruent than congruent outcomes in a time window ranging from around 2200 to 2690 ms (BF_10_ between 3 and 118.02, see Figure 3). This indicates that 9‐month‐old infants were surprised to see the agent searching in the empty location when she knew where the object really was (True Belief). The average pupil dilation did not differ between congruent and incongruent outcomes (True Belief: BF_10_ = 0.15, congruent: *N *= 46, *M *= 0.003, *SD *= 0.031; incongruent: *N *= 42, *M *= 0.005, *SD *= 0.024).

#### 4.1.2 Eighteen‐Month‐Old Infants

The 18‐month‐old infants showed evidence against differential pupil dilation depending on the outcome both in the False Belief and the True Belief condition in the Action Prediction block (dynamic analysis: see Figure 3; average across outcome phase: False Belief: BF_10_ = 0.15, congruent: *N *= 52, *M *= 0.009, *SD *= 0.026; incongruent: *N *= 49, *M *= 0.011, *SD *= 0.032; True Belief: BF_10_ = 0.26, congruent: *N *= 49, *M *= 0.011, *SD *= 0.026; incongruent: *N *= 53, *M *= 0.015, *SD *= 0.025). Thus, 18‐month‐old infants’ showed no sign of correct action prediction, neither when the agent held a true nor a false belief.

In neither of the age groups, infants’ pupil response (in the Object Memory and Action Prediction block) was related to their joint attention scores, MSR (only acquired at 18‐month‐old infants), or their age in days (see Supporting Information S14–S16). Highly similar results were obtained in within‐subject analyses for both age groups (see Supporting Information S17).

##### 4.1.2.1 Action Prediction—Anticipatory Looking

In anticipation of the action outcome, 18‐month‐old infants looked more to the current object location than to the empty location both in False Belief trials (Wilcoxon test for DLS > 0: BF_10_ = 9.68; *W *= 639; $\hat{R}$ = 1.007, *N *= 51; *M *= 0.32; *SD *= 0.76; binomial test first for first look: BF_10_ = 1.72, details see Supporting Information S13) and in True Belief trials (DLS > 0: BF_10_ = 3.01; *W *= 460.5; $\hat{R}$ = 1.001; *N *= 48; *M *= 0.28; *SD *= 0.76, first look: BF_10_ = 10.46, see Supporting Information S13). This indicates that infants expected the agent to search where the object really was, independently of her belief. As before, 9‐month‐old infants showed no systematic anticipatory looking (see Supporting Information S13).

Nine‐ and 18‐month‐old infants’ pupil response in the Object Memory block was not predictive of their pupil dilation in the Action Prediction block, nor was anticipatory looking related in the two blocks (see Supporting Information S18).

## 5 Discussion

According to the altercentric theory, young infants prioritize the encoding of events witnessed together with others over events witnessed alone (Southgate 2020). In case a change occurs in the absence of others, infants may therefore misremember scenes the way the others saw them last (Grosse Wiesmann and Southgate 2021; Southgate 2020). Here we tested this theory empirically, and we indeed found that 9‐month‐old infants seem to misremember an object's location based on where the other had seen it last and not where it had really moved in the meantime. This was indicated by larger pupil dilation to reveal the object in its current location. Strikingly, this altercentric memory bias had vanished by 18 months, by which age infants seemed to remember the object location correctly. The altercentric bias observed at 9 months was not related to correctly predicting where the agent would reach for the object.

Why should young infants misremember objects where others represent them to be? While leading to memory errors in specific situations, namely, when another agent has a false belief, prioritized encoding of what others’ have seen is highly beneficial for central tasks that infants face. During infancy, the ability to learn from others is paramount for survival and development. Young infants need to orient themselves and learn from a highly complex environment. In doing so, they heavily rely on others. The observed altercentric bias may provide young infants with a way of considering what others’ attend and refer to (Southgate 2020; Tebbe et al. 2024). This may allow infants to learn from others and use them as guidance when processing their surroundings. Representing the world as seen by others may also allow young infants to consider their perspectives despite limited processing capacities (Grosse Wiesmann and Southgate 2021; Tebbe et al. 2024). Especially, given infants’ own restricted ability to interact with the world, the objects of others’ attention and communication, and capacity to predict others’ actions, may be of greater significance to young infants than their own correct memory of their surroundings. As such, the altercentric bias observed in the first year of life could serve as an adaptive mechanism in infancy to facilitate both learning and social cognition, facilitating essential aspects of early cognitive development.

In the second year of life, in turn, given infants’ growing capacity to engage with their surroundings, the importance of a correct representation of their physical environment increases and the reliance on others begins to decrease. Against this background, our finding that the altercentric bias had vanished by 18 months, giving way for accurate object memory, seems highly adaptive. This finding also yields an explanation for why Manea et al. (2023) neither found an altercentric memory error nor correct object memory at 12 months. Possibly, at this age, infants transition from prioritizing information seen by others to prioritizing their own view, and this conflict between the others’ and infants’ own perspective may have led to the null‐finding at 12 months in Manea et al. (2023). Indeed, it has been argued that, as infants become more able to act on their environment and their self‐awareness develops, infants’ own perspective may become dominant over that of others (Grosse Wiesmann et al. 2024; Grosse Wiesmann and Southgate 2021; Southgate 2020). Supporting this notion, in the domain of memory, a recent study found that infants who did not yet recognize themselves in the mirror remembered items better that were relevant to others while mirror self‐recognizers remembered self‐relevant items better similar to older children and adults (Grosse Wiesmann et al. 2024). Further, conflict processing was enhanced in mirror self‐recognizers when watching scenes where an agent had a perspective conflicting with that of the infant (Yeung et al. 2022). Thus, at 18‐month infants may still experience a conflict between prioritizing self‐ and other‐relevant content, but our findings suggest that by this age the self‐perspective dominates. In our study, evidence for a relation between object memory and developing self‐awareness as measured by the MSR task was inconclusive due to insufficient power. Future research will need to follow up on whether the observed transition from altercentric to correct object memory is related to the emergence of self‐awareness and to explore individual differences in pupil responses in relation to infants’ MSR.

In sum, our findings of an altercentric memory error at 9 months that seems to vanish by 18 months are fully in line with theoretical suggestions by the altercentric theory (Southgate 2020). A further prediction of the altercentric theory is that altercentric memory errors, as observed in our study, may be at the basis of infants’ correct predictions of the actions of agents with a false belief (Grosse Wiesmann and Southgate 2021; Southgate 2020). Contrary to this suggestion, we did not find a relation between infants’ altercentric memory errors in the Object Memory block and their action prediction in the Action Prediction block. However, in this context, it is important to note that, in our paradigm, infants did not show any correct action prediction when the agent had a false belief. This was neither the case in infants’ pupil in response to an inconsistent action nor in their anticipatory looking before the agent's action was shown. In fact, 18‐month‐old infants expected the agent to reach to where the object really was both when she had a true or a false belief. This is in line with numerous other non‐replications of early false belief‐based action prediction at this age group (Grosse Wiesmann et al. 2018; Kampis et al. 2021; Poulin‐Dubois and Yott 2018). These non‐replications suggest that infants do not robustly predict others’ actions based on their beliefs. Our study differed in layout and set‐up from previous infant false beliefs and was not optimized to elicit expectations about an agent's future actions (e.g., Behne et al. 2005). Two recent studies also examined the robustness of infants’ action anticipation in the context of goal attribution more generally (Ganglmayer et al. 2019; Sirois et al. 2023). Interestingly, these findings did not support the interpretation of consistent goal attribution in infants using looking behavior (Ganglmayer et al. 2019) or pupillometry (Sirois et al. 2023), but suggest that infants rather seem to anticipate the movement pattern of an action than that they flexibly attribute action goals. Although our findings indicate that young infants misremembered objects as seen by others, this bias does not seem to help them to predict others’ actions. Rather, our findings suggest that the altercentric bias might serve a different function than aiding action prediction. Beyond action prediction, an altercentric bias may also allow infants to use the attention of others to guide them in a complex environment. That is, they primarily encode what others attend to, helping them to reduce information to what is relevant to others (e.g., Grosse Wiesmann et al. 2024). Further, prioritizing the targets of others’ attention may help identify the referents of their communication (e.g., Tebbe et al. 2024). As such, the altercentric bias may play an important role as an adaptive and efficient social learning mechanism early in life.

Notably, 9‐month‐old infants did not show higher pupil dilation in response to reality incongruent outcomes in True Belief trials of the Object Memory task. Recent research indicates that infants do not form strong expectations about objects when an agent with a true belief is present (Manea et al. 2023). Similarly, other implicit ToM tasks have revealed chance level performance in True Belief trials (e.g., Crivello and Poulin‐Dubois 2018; Poulin‐Dubois and Chow 2009; Priewasser et al. 2018; Rubio‐Fernández 2019). In our study, 9‐month‐old infants were not surprised to see the object revealed in the reality‐incongruent location in True Belief trials, but they remembered the object's location in the action prediction block where they expected the agent to reach into the correct location in the True Belief trials. This indicates that infants encoded and recalled where the object was, but that this may have been more relevant in the action prediction context than when the object itself was revealed. This fits with the idea that, in the presence of others, young infants may prioritize what is relevant to the other over their own perspective. In a context where the other is likely to act on an object, it might therefore be more important to encode information about this object. A recent study suggests that this is not only the case for the objects’ location but also their features (Grosse Wiesmann et al. 2024). In contrast, 18‐month‐old infants remembered the object's location both in the false belief and true belief context, regardless of its relevance to the other agent. Specifically, they looked more toward the side where the object was located both when the object was revealed (in the Object Memory block) and when the agent acted on the object (in the Action Prediction block). These findings for the True Belief condition match the development observed for the False Belief situation, where infants only seem to encode what is relevant to others in the first year of life, but then shift to prioritizing their own view by 18 months. Indeed, anticipatory looking results indicate that 18‐month‐old infants preferentially fixated the object's current location, even preceding the agent's action when she had a false belief. That is, they did not anticipate her false belief‐based actions correctly. The extent to which anticipatory looking measures are suitable to assess spontaneous (goal‐directed) action prediction in Theory of Mind tasks is a matter of ongoing debate (see Barone et al. 2019; Powell et al. 2018; Schuwerk et al. 2018). The inconsistencies in the literature underscore the need for further research and large‐scale studies in multi‐lab collaborations such as the ManyBabies consortium (e.g., Schuwerk et al. 2021) potentially in combination with multi‐analytical approaches (see Calignano et al. 2024; Sirois et al. 2023). Pupillometry offers a powerful means for capturing the temporal dynamics of cognitive processing (Hepach 2024). This is particularly valuable in developmental research, where such tracking is difficult to achieve with methods based on aggregated and cumulative measures such as looking time. As the choices made during the preprocessing stage can significantly influence the presence and direction of effects (see Calignano et al. 2024; Sirois et al. 2023), we analyzed our data using different preprocessing parameters and showed that the results were robust with respect to the choice of filters, baseline correction window, and inclusion rate (i.e., minimum number of sample points in the outcome phase).

Relatedly, it is an open question how infants’ encoding of an object's relocation differs between true versus false belief scenarios. While the observed altercentric bias indicates that infants remember events better that they witness together with another person, it is not necessarily the case that infants experience the situation as co‐witnessed. Alternatively, it is also possible that the mere presence of another agent, or their gaze‐cueing of an event, leads to better memory of this event and thus, in false belief situations like in the present paradigm, to an altercentric memory bias. Future experiments should investigate whether the mere presence of another agent influences infants’ encoding of the object's position, even if the agent does not observe the transfer. Alternatively, active engagement by the agent, for example, ostensive social cues, might be necessary to enhance infants’ memory of the object's location. In addition, future studies should explore whether infants encode the object's position in a person‐specific manner. Examining how different individuals influence infants’ expectations or memory of the object's location would shed light on whether to the observed memory bias is related to the specific agent that had been present or whether it is independent of a specific person and merely enhanced by the presence or gaze of any person during the hiding event. Prior research suggests that infants do not bind preferences to a specific agent, but overgeneralize them across individuals (Kampis et al. 2013). Further, supporting the notion that the observed memory bias is not agent‐specific, a similar altercentric bias was found when the agent was no longer present in the outcome phase (Manea et al. 2023).

In addition, future research should specify the decrease of the altercentric bias by sampling its developmental trajectory longitudinally across the first 2 years of life, also in relation to the development of a self‐concept and increasing agency (e.g., Bednarski et al. 2022). As previous research has pointed to cross‐cultural variation in when children acquire a self‐concept and recognize themselves in the mirror (Broesch et al. 2011; Cebioğlu and Broesch 2021), the development of altercentric biases might also vary significantly across different cultural contexts. Further, it is an open question how the receding altercentric bias in infancy relates to altercentric influences observed in older children (Speiger et al. 2024) and adults (Furlanetto et al. 2016; Kovács et al. 2010; Samson et al. 2010; Speiger et al. 2025; Van Der Wel et al. 2014). A recent study found that both infants and adults neurally processed the perspective of others like their own perception, suggesting a similar neural mechanism of altercentric processing in infants and adults (Tebbe et al. 2024). Future research will need to investigate how this bias develops between infancy and adulthood, and how it relates to verbally reasoning about others’ beliefs (Speiger et al., 2024).

Taken together, in line with the altercentric theory (Southgate 2020), our study provides evidence for an altercentric bias in the first year of life, leading to memory errors in situations where another agent has a conflicting perspective. This bias seems to recede between the first and second year of life, and by 18 months, infants show correct object memory, independently of the perspective of others. In contrast to theoretical proposals (Grosse Wiesmann and Southgate 2021; Southgate 2020), this altercentric bias does not seem to primarily serve as a tool for predicting others’ behavior. Rather, we suggest that it might aid infants in selecting and prioritizing the most relevant information by using the attention of others as orientational cue and learning opportunity in a complex environment. Contrary to the traditional view, this questions long‐held assumptions that infants perceive their world solely from their own perspective. Far from being egocentric, as argued by Piaget (1926), our findings indicate that infants instead may start out altercentric. Our study suggests that, only later, as infants become more capable of actively engaging with and influencing their environment, they develop the egocentric stance known in the toddler years.

## Ethics Statement

The study was approved by the *Ethics* Advisory *Board* at the University of Leipzig.

## Conflicts of Interest

The authors declare that they have no competing interests.

## Supporting information



Supporting Information

## Data Availability

Data in accordance with data protection regulations will be made available before acceptance.

## Endnotes

1 We refer to these trials as False Belief and True Belief trials as this describes the agent's belief state. This does not imply that infants had to process these beliefs to form expectations about the outcome.
