# Supplementary material for: Altercentric Memory Error at 9 Months But Correct Object Memory by 18 Months Revealed in Infants’ Pupil
Source: Dev Sci. 2025 Mar 30;28(3):e70016. doi: 10.1111/desc.70016 (PMC11955748; doi:10.1111/desc.70016)
Supplement: Supplementary file 1 — Supporting Information [file DESC-28-e70016-s001.docx]

**Supplementary Material for the manuscript ‘Altercentric memory error at 9 months but correct object memory by 18 months revealed in infants’ pupil’**

Anna-Lena Tebbe, Katrin Rothmaler, Hannah Elena Zielke, Robert Hepach, & Charlotte Grosse Wiesmann

**Authors**

**Anna-Lena Tebbe** (corresponding author)

Research Group ‘Milestones of Early Cognitive Development’, Max Planck Institute for Human Cognitive and Brain Sciences, Leipzig, Germany

tebbe@cbs.mpg.de

ORCID: 0000-0003-4933-2797

**Katrin Rothmaler**

Research Group ‘Milestones of Early Cognitive Development’, Max Planck Institute for Human Cognitive and Brain Sciences, Leipzig, Germany

ORCID: 0000-0001-8588-7664

**Hannah Elena Zielke**

University of Leipzig, Germany

h.zielke@studserv.uni-leipzig.de

ORCID: 0009-0007-9240-5046

**Robert Hepach**

Department of Experimental Psychology, University of Oxford, UK

robert.hepach@psy.ox.ac.uk

ORCID: 0000-0003-4780-6549

**Charlotte Grosse Wiesmann**

Research Group ‘Milestones of Early Cognitive Development’, Max Planck Institute for Human Cognitive and Brain Sciences, Leipzig, Germany

and

Cognitive Neuroscience Lab, Department of Liberal Arts and Sciences, University of Technology Nuremberg, Germany

wiesmann@cbs.mpg.de

ORCID: 0000-0003-2577-6174

**Contents**

S1. Sequential analysis

S2. Overview of pseudo-randomized orders

S3. Control analysis for effects of order

S4. Results for 50% inclusion criteria

S5. Robustness check

S6. Control analysis for gaze points during change of location

S7. Flow chart of sample size

S8. Control analysis for missing data points

S9. Visualization of gaze points

S10. Q-Q plots

S11. Baseline-corrected pupil dilation averages

S12. Results for LMM with Belief x Outcome interaction

S13. Anticipatory looking

S14. Relation with Joint attention score

S15. Relation with Mirror Self-recognition

S16. Relation with age

S17. Results for within-subject analysis

S18. Relation of altercentric bias with action prediction

**S1. Sequential analysis**

We continued data collection until we a) had collected a minimal sample size of *N* = 25 for each age group and block (i.e., Object Memory, Action Prediction) and b) had gathered moderate evidence for or against an effect in all four critical comparisons in False Belief trials (Bayes Factor BF_10_>3 or <1/3). Accordingly, data collection was continued until the BF of all critical comparisons were stable (defined as 5 children consecutively tested with BF_10_>3 or <1/3), resulting in a total sample of *N* = 58 9-months-olds. For the 18-months-olds, our sample converged at *N* = 29, but due to complex preprocessing, testing was continued until *N* = 62.

**Pupil Dilation Results at convergence of sequential Bayes analyses**

**Object Memory**

*Nine-months-olds.* The results at convergence (*N* = 58) were qualitatively similar to those in the full sample (reported in the Results section). Specifically, at *N* = 58, 9-months-olds’ pupil size was larger during the reality-congruent than the reality-incongruent outcome in False Belief trials when averaging across the entire 5 second time window (BF_10_ = 15.73; congruent: *N* = 49, *M*= .018, *SD* = .03; incongruent: *N* = 44, *M* = .003, *SD* = .017, see Fig. S1). In contrast, the BF_10_ testing for differences in the pupil dilation in the True Belief control condition revealed no such difference, BF_10_ = .24, congruent: *N* = 29, *M* = .012, *SD* = .024; incongruent: *N* = 39, *M* = .02, *SD* = .027.

*Eighteen-months-olds.* The results at convergence (*N* = 29) were qualitatively similar to those in the full sample (see Results). Specifically, at *N* = 29, the average pupil dilation across the outcome time window after object appearance, resulted in moderate evidence against a difference for False Belief trials (BF_10_ = .26, congruent: *N* = 27, *M* = .014, *SD* = .023; incongruent: *N* = 25, *M* = .019, *SD* = .027, see Fig. S1). The LMM comparing the averaged pupil response in the True Belief control condition yielded inconclusive evidence (BF_10_ = .40, congruent: *N* = 20, *M* = .017, *SD* = .022; incongruent: *N* = 13, *M* = .025, *SD* = .026).

**Action Prediction**

*Nine-months-olds.* For *N* = 58, the 9-months-olds showed evidence against differential pupil dilation in the False Belief trials, BF_10_ = .33, congruent: *N* = 45, *M* = .012, *SD* = .028; incongruent: *N* = 47, *M* = .007, *SD =* .025, see Fig. S1. The Bayesian t-test comparing the averaged pupil dilation to congruent and incongruent outcomes in True Belief trials showed moderate evidence against outcome differences (BF_10_ = .16, congruent: *N* = 37, *M* = .006, *SD* = .029; incongruent: *N* = 37, *M* = .009, *SD* = .024).

*Eighteen-months-olds.* At convergence (*N* = 29), the 18-months-olds showed moderate evidence against differential pupil dilation depending on the outcome in the False Belief condition, i.e., 18-months-olds’ pupil size increased similarly for both the congruent and the incongruent outcome in False Belief (BF_10_ = .21, congruent: *N* = 26, *M* = .016, *SD* = .025; incongruent: *N* = 21, *M* = .013, *SD =* .039, see Fig. S1). Similarly, the Bayesian t-test comparing the averaged pupil dilation to congruent and incongruent outcomes in True Belief trials showed moderate evidence against outcome differences (BF_10_ = .23, congruent: *N* = 22, *M* = .015, *SD* = .027; incongruent: *N* = 21, *M* = .018, *SD* = .026).


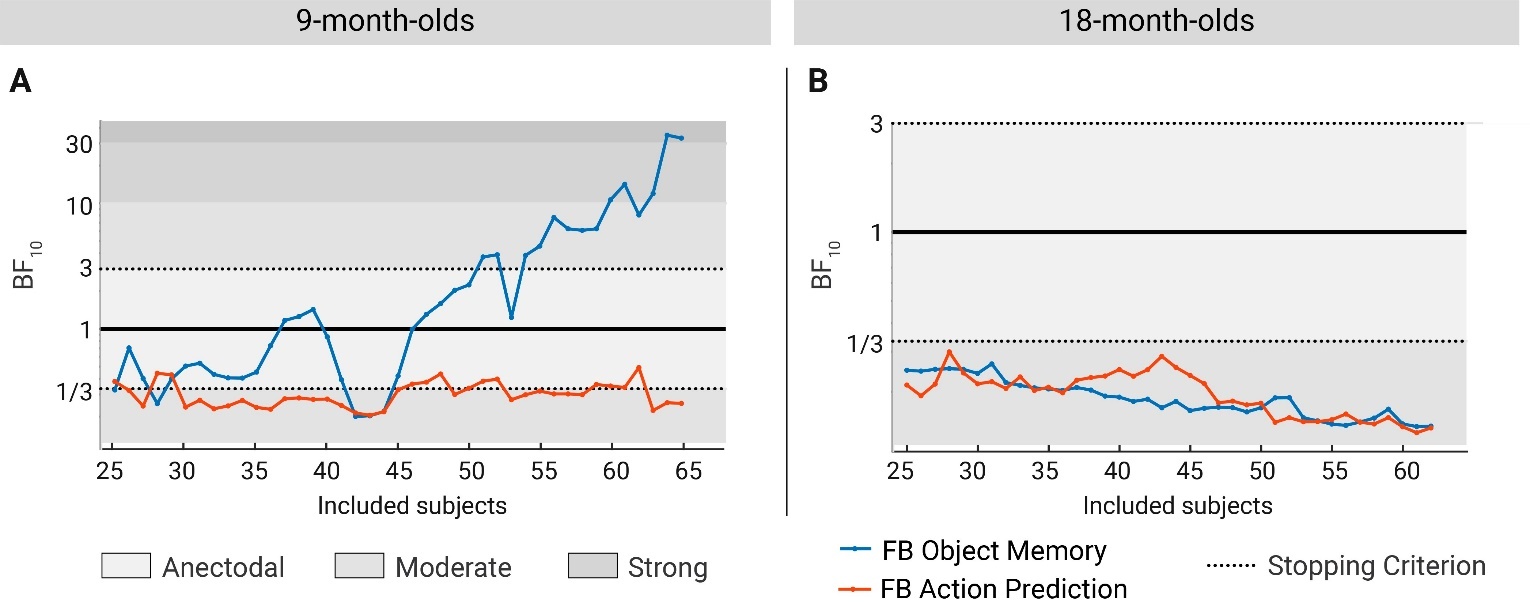


*Figure S1.* Figure S1 shows the sequential development of the BF_10_ for our preregistered analyses for the Object Memory False Belief trials (blue), and the Action Prediction False Belief trials (orange) for the 9-month-olds (A) and 18-month-olds (B). Data collection was stopped when all BFs of the preregistered critical comparisons were stable (defined as 5 children consecutively tested with BF_10_>3 or <1/3), and showed moderate evidence for or against a difference between the reality congruent and reality incongruent False Belief outcomes. We collected a minimum N of 25 participants.

**S2. Overview of pseudo-randomized presentation orders**

| Order | 1^st^ trial | 2^nd^ trial | 3^rd^ trial | 4^th^ trial |
| --- | --- | --- | --- | --- |
| Order 1 | TB congruent | TB incongruent | FB congruent | FB incongruent |
| Order 2 | TB incongruent | TB congruent | FB incongruent | FB congruent |
| Order 3 | FB congruent | FB incongruent | TB congruent | TB incongruent |
| Order 4 | FB incongruent | FB congruent | TB incongruent | TB congruent |
| Order 5 | TB congruent | FB congruent | TB incongruent | FB incongruent |
| Order 6 | TB incongruent | FB incongruent | TB congruent | FB congruent |
| Order 7 | FB congruent | TB congruent | FB incongruent | TB incongruent |
| Order 8 | FB incongruent | TB incongruent | FB congruent | TB congruent |

*Table S1*. Overview of the eight pseudorandomized orders (first column) implemented in the paradigm. Each of the two blocks (Object Memory, Action Prediction) consisted of four trials, so that a child saw 8 trials in total (see rows). Note that the trial order was the same in both blocks. Four orders started with a True Belief (TB) trial or a False Belief (FB) trial, respectively. In each order, there were two trials per block in which the object entered the left location first and two trials in which the object entered the right location first, ensuring that i) the reality congruent and ii) the presented outcome were on either side equally often.

**S3. Control analysis for effects of order**

S3.1. Block Order

S3.2. First Trial Congruency

**S3.1. Block Order (Object Memory vs Action Prediction as first block)**

To test whether infants’ pupil response was a function of which block was presented first, we ran control analysis to investigate potential effects of the block order on infants’ pupil dilation. We conducted LMMs using the average pupil dilation (PD) as the dependent variable, *Outcome* and *Block Order* (Object Memory vs Action Prediction block first) as independent variables and a random intercept per subject.

**Object Memory**

*Nine-months-olds.* For the Object Memory block, there was a main effect of *Outcome* for 9-months-olds in the False Belief trials, BF_10_ = 21.43 (see Table S2), with greater pupil dilation to reality congruent outcomes in False Belief trials as reported in the main manuscript. There was no indication of a main effect of *Block Order* or the interaction of *Outcome* and *Block Order* (BF_10_ = 1.66, and BF_10_ = 1.4, respectively). In the True Belief control condition, there was moderate evidence against main effects (*Outcome*, BF_10_ = .313; *Block Order*, BF_10_ = .286), and against an interaction of the factors (BF_10_ = .309, see Table S3). This indicates that there was no effect of trial order on the Object Memory block.

*Eighteen-months-olds.* For 18-months-olds the LMM showed moderate evidence against a main effect of *Outcome* (BF_10_ = .136), *Block Order* (BF_10_ = .189), and inconclusive evidence concerning an interaction (BF_10_ = .371, see Table S2) in the Object Memory False Belief trials. In the True Belief control condition, our analysis revealed moderate evidence against main effects of *Outcome* (BF_10_ = .182) and *Block Order* (BF_10_ = .243), and inconclusive evidence concerning an interaction (BF_10_ = .573, see Table S3), indicating that there was no effect of trial order.

**Object Memory**

**False Belief – Block Order**

|  | | Model 0 (1st row) and Model 1 (2nd row) |  | |
| --- | --- | --- | --- | --- |
|  |  |  | BF | BF_10_ |
| 9 months (*N* = 73) | ME *Outcome* | PD ~ ID + Outcome + BO | 24.27 | 21.43 |
|  |  | PD ~ ID + BO | 1.13 |  |
|  | ME *Block Order* | PD ~ ID + Outcome + BO | 24.27 | 1.67 |
|  |  | PD ~ ID + Outcome | 14.57 |  |
|  | Interaction | PD ~ ID + Outcome + BO + Outcome* BO | 34.05 | 1.40 |
|  |  | PD ~ ID + Outcome + BO | 24.27 |  |
| 18 months (*N* = 62) | ME *Outcome* | PD ~ ID + Outcome + BO | .026 | .136 |
|  |  | PD ~ ID + BO | .194 |  |
|  | ME *Block Order* | PD ~ ID + Outcome + BO | .026 | .189 |
|  |  | PD ~ ID + Outcome | .140 |  |
|  | Interaction | PD ~ ID + Outcome + BO + Outcome* BO | .010 | .371 |
|  |  | PD ~ ID + Outcome + BO | .026 |  |

*Table S2.* Table S2 shows the null model (Model 0) and the alternative model (Model 1) for different model comparisons with the respective Bayes Factors of the models (BF) and model comparisons (BF_10_) for the Object Memory False Belief trials in 9-months-olds (*N* = 73) and 18-months-olds (*N* = 62) and their potential association with *Block Order*. Abbreviations: ME = Main Effect, PD = averaged Pupil Dilation response, ID = subject intercept, BO = Block Order.

**Object Memory**

**True Belief – Block Order**

|  | | Model 0 (1st row) and Model 1 (2nd row) |  | |
| --- | --- | --- | --- | --- |
|  |  |  | BF | BF_10_ |
| 9 months (*N* = 57) | ME *Outcome* | PD ~ ID + Outcome + BO | .092 | .313 |
|  |  | PD ~ ID + BO | .293 |  |
|  | ME *Block Order* | PD ~ ID + Outcome + BO | .092 | .286 |
|  |  | PD ~ ID + Outcome | .321 |  |
|  | Interaction | PD ~ ID + Outcome + BO + Outcome* BO | .028 | .309 |
|  |  | PD ~ ID + Outcome + BO | .092 |  |
| 18 months (*N* = 53) | ME *Outcome* | PD ~ ID + Outcome + BO | .045 | .182 |
|  |  | PD ~ ID + BO | .247 |  |
|  | ME *Block Order* | PD ~ ID + Outcome + BO | .045 | .243 |
|  |  | PD ~ ID + Outcome | .184 |  |
|  | Interaction | PD ~ ID + Outcome + BO + Outcome* BO | .026 | .573 |
|  |  | PD ~ ID + Outcome + BO | .045 |  |

*Table S3.* Model comparisons for the Object Memory True Belief trials in 9-months-olds (*N* = 57) and 18-months-olds (*N* = 53) and their association with Block Order. Abbreviations: ME = Main Effect, PD = averaged Pupil Dilation response, ID = subject intercept, BO = Block Order.

**Action Prediction**

*Nine-months-olds.* For the Action Prediction block*,* an LMM in 9-months-olds revealed strong evidence for a main effect of *Block Order* in False Belief trials, BF_10_ = 11.24, . Specifically, the average pupil response in False Belief Action Prediction trials was greater when the Object Memory block was presented first compared to when infants were presented with the Action Prediction block first. There was no indication for an effect of Block Order in True Belief trials, BF_10_ = .361. The LMM revealed no effect of *Outcome* (False Belief: BF_10_ = .226, True Belief: BF_10_ = .147), and no interaction of *Block Order* and *Outcome*, (False Belief: BF_10_ = .190, True Belief: BF_10_ = .233, see Table S4 & S5).

*Eighteen-months-olds.* For 18-months-olds the LMM showed moderate evidence against a main effect of *Outcome* (False Belief: BF_10_ = .146; True Belief: BF_10_ = .270, see Table S4 & S5), and against an interaction effect of *Outcome* and *Block Order* (False Belief: BF_10_ = .223; True Belief: BF_10_ = .212) in the Action Prediction block. In addition, there was moderate evidence against a main effect of *Block Order* in True Belief trials (BF_10_ = .223)and False Belief (BF_10_ = .285).

**Action Prediction**

**False Belief – Block Order**

|  | | Model 0 (1st row) and Model 1 (2nd row) |  | |
| --- | --- | --- | --- | --- |
|  |  |  | BF | BF_10_ |
| 9 months (*N* = 65) | ME *Outcome* | PD ~ ID + Outcome + BO | 3.01 | .226 |
|  |  | PD ~ ID + BO | 13.31 |  |
|  | ME *Block Order* | PD ~ ID + Outcome + BO | 3.01 | 11.24 |
|  |  | PD ~ ID + Outcome | .268 |  |
|  | Interaction | PD ~ ID + Outcome + BO + Outcome* BO | .572 | .190 |
|  |  | PD ~ ID + Outcome + BO | 3.01 |  |
| 18 months (*N* = 62) | ME *Outcome* | PD ~ ID + Outcome + BO | .043 | .146 |
|  |  | PD ~ ID + BO | .152 |  |
|  | ME *Block Order* | PD ~ ID + Outcome + BO | .043 | .285 |
|  |  | PD ~ ID + Outcome | .152 |  |
|  | Interaction | PD ~ ID + Outcome + BO + Outcome* BO | .010 | .223 |
|  |  | PD ~ ID + Outcome + BO | .043 |  |

*Table S4.* Model comparisons for the Action Prediction False Belief trials in 9-months-olds (*N* = 65) and 18-months-olds (*N* = 62) and their association with Block Order. Abbreviations: ME = Main Effect, PD = averaged Pupil Dilation response, ID = subject intercept, BO = Block Order.

**Action Prediction**

**True Belief – Block Order**

|  | | Model 0 (1st row) and Model 1 (2nd row) |  | |
| --- | --- | --- | --- | --- |
|  |  |  | BF | BF_10_ |
| 9 months (*N* = 59) | ME *Outcome* | PD ~ ID + Outcome + BO | .053 | .147 |
|  |  | PD ~ ID + BO | .358 |  |
|  | ME *Block Order* | PD ~ ID + Outcome + BO | .053 | .361 |
|  |  | PD ~ ID + Outcome | .146 |  |
|  | Interaction | PD ~ ID + Outcome + BO + Outcome* BO | .012 | .233 |
|  |  | PD ~ ID + Outcome + BO | .053 |  |
| 18 months (*N* = 58) | ME *Outcome* | PD ~ ID + Outcome + BO | .059 | .270 |
|  |  | PD ~ ID + BO | .217 |  |
|  | ME *Block Order* | PD ~ ID + Outcome + BO | .059 | .223 |
|  |  | PD ~ ID + Outcome | .263 |  |
|  | Interaction | PD ~ ID + Outcome + BO + Outcome* BO | .012 | .212 |
|  |  | PD ~ ID + Outcome + BO | .059 |  |

*Table S5.* Model comparisons for the Action Prediction True Belief trials in 9-months-olds (*N* = 59) and 18-months-olds (*N* = 58) and their association with Block Order. Abbreviations: ME = Main Effect, PD = averaged Pupil Dilation response, ID = subject intercept, BO = Block Order.

**S3.2. First Trial congruency**

To further explore potential order effects, we investigate whether the order of outcome congruency of the first trial had an effect on the pupil dilation. To this end, we conducted LMMs using the average pupil dilation (PD) as the dependent variable, *Outcome* and *First Trial Congruency* as independent variables and a random intercept per subject.

**Object Memory**

*Nine-months-olds.* The analysis of the Object Memory block showed strong evidence for a main effect of *Outcome* in False Belief (BF_10_ = 16.08, supporting our findings reported in the main manuscript), but not in True Belief trials (BF_10_ = .316). There was no indication of a main effect of *First Trial Congruency* (False Belief*:* BF_10_ = .21; True Belief: BF_10_ = .207), and moderate evidence against an interaction of the two factors (False Belief: BF_10_ = .186; True Belief: BF_10_ = .310, see Table S6 & S7).

*Eighteen-months-olds.* For 18-months-olds the LMM showed moderate evidence against a main effect of *Outcome* (False Belief: BF_10_ = .126; True Belief: BF_10_ = .188). In addition, we found moderate evidence against an interaction in False Belief trials (BF_10_ = .197), and no indication of a main effect of *First Trial Congruency* (False Belief: BF_10_ = .349; True Belief: BF_10_ = .273, see Table S6 & S7). In True Belief trials, the BF_10_ testing for an interaction was inconclusive, BF_10_ = 1.97.

**Object Memory**

**False Belief – First Trial Congruency**

|  | | Model 0 (1^st^ row) and Model 1 (2^nd^ row) |  | |
| --- | --- | --- | --- | --- |
|  |  |  | BF | BF_10_ |
| 9 months  (*N* = 73) | ME *Outcome* | PD ~ ID + Outcome + First Trial | 3.06 | 16.08 |
|  |  | PD ~ ID + First Trial | .19 |  |
|  | ME *First Trial* | PD ~ ID + Outcome + First Trial | 3.06 | .21 |
|  |  | PD ~ ID + Outcome | 14.54 |  |
|  | Interaction | PD ~ ID + Outcome + First Trial + Outcome* First Trial | .567 | .186 |
|  |  | PD ~ ID + Outcome + First Trial | 3.06 |  |
| 18 months  (*N* = 62) | ME *Outcome* | PD ~ ID + Outcome + First Trial | .048 | .126 |
|  |  | PD ~ ID + First Trial | .38 |  |
|  | ME *First Trial* | PD ~ ID + Outcome + First Trial | .048 | .349 |
|  |  | PD ~ ID + Outcome | .137 |  |
|  | Interaction | PD ~ ID + Outcome + First Trial + Outcome* First Trial | .009 | .197 |
|  |  | PD ~ ID + Outcome + First Trial | .048 |  |

*Table S6.* Table S6 shows the null model (Model 0) and the alternative model (Model 1) for different model comparisons with the respective Bayes Factors of the models (BF) and model comparisons (BF_10_) for False Belief trials of the Object Memory block in 9-months-olds (*N* = 73) and 18-months-olds (*N* = 62) for potential order effects (*First Trial Congruency*). Abbreviations: ME = Main Effect, PD = averaged Pupil Dilation response, ID = subject intercept.

**Object Memory**

**True Belief – First Trial Congruency**

|  | | Model 0 (1^st^ row) and Model 1 (2^nd^ row) |  | |
| --- | --- | --- | --- | --- |
|  |  |  | BF | BF_10_ |
| 9 months  (*N* = 57) | ME *Outcome* | PD ~ ID + Outcome + First Trial | .068 | .316 |
|  |  | PD ~ ID + First Trial | .214 |  |
|  | ME *First Trial* | PD ~ ID + Outcome + First Trial | .068 | .207 |
|  |  | PD ~ ID + Outcome | .326 |  |
|  | Interaction | PD ~ ID + Outcome + First Trial + Outcome* First Trial | .021 | .31 |
|  |  | PD ~ ID + Outcome + First Trial | .068 |  |
| 18 months  (*N* = 53) | ME *Outcome* | PD ~ ID + Outcome + First Trial | .049 | .188 |
|  |  | PD ~ ID + First Trial | .26 |  |
|  | ME *First Trial* | PD ~ ID + Outcome + First Trial | .049 | .273 |
|  |  | PD ~ ID + Outcome | .179 |  |
|  | Interaction | PD ~ ID + Outcome + First Trial + Outcome* First Trial | .096 | 1.97 |
|  |  | PD ~ ID + Outcome + First Trial | .049 |  |

*Table S7.* Model comparisons for True Belief trials of the Object Memory block in 9-months-olds (*N* = 57) and 18-months-olds (*N* = 53), testing for potential order effects (First Trial Congruency). Abbreviations: ME = Main Effect, PD = averaged Pupil Dilation response, ID = subject intercept.

**Action Prediction**

*Nine-months-olds.* For the Action Prediction block*,* we observed moderate evidence against a main effect of *Outcome* for 9-months-olds (False Belief trials: BF_10_ = .223; True Belief trials: BF_10_ = .131), and no *Outcome* and *First Trial Congruency* interaction (False Belief: BF_10_ = .225; True Belief: BF_10_ = .234, see Table S8 & S9). There was moderate evidence against a main effect of *First Trial Congruency* in True Belief trials*,* (BF_10_ = .236; inconclusive evidence for False Belief trials, BF_10_ = .356). This suggests that the congruency of the first trial had no major effects on 9-months-olds pupil response in the Action Prediction block.

*Eighteen-months-olds.* For 18-months-olds, there was moderate evidence against a main effect of *Outcome*, (False Belief: BF_10_ = .16; True Belief: BF_10_ = .286). The LMM showed no evidence for interaction effects, (False Belief: BF_10_ = .287, True Belief: BF_10_ = .212, see Table S8 & S9). For True Belief trials, we observed moderate evidence against a main effect of *First Trial Congruency*, BFs_10_ = .292. The Bayes Factor in False Belief trials was inconclusive (BF_10_ = .772). Accordingly, the order of congruent and incongruent outcome had no systematic effects on the pupil recorded in the Action Prediction block in the 18-month-olds.

**Action Prediction**

**False Belief – First Trial Congruency**

|  | | Model 0 (1st row) and Model 1 (2nd row) |  | |
| --- | --- | --- | --- | --- |
|  |  |  | BF | BF_10_ |
| 9 months  (*N* = 65) | ME *Outcome* | PD ~ ID + Outcome + First Trial | .096 | .223 |
|  |  | PD ~ ID + First Trial | .433 |  |
|  | ME *First Trial* | PD ~ ID + Outcome + First Trial | .096 | .356 |
|  |  | PD ~ ID + Outcome | .27 |  |
|  | Interaction | PD ~ ID + Outcome + First Trial + Outcome* First Trial | .022 | .225 |
|  |  | PD ~ ID + Outcome + First Trial | .096 |  |
| 18 months  (*N* = 62) | ME *Outcome* | PD ~ ID + Outcome + First Trial | .116 | .16 |
|  |  | PD ~ ID + First Trial | .725 |  |
|  | ME *First Trial* | PD ~ ID + Outcome + First Trial | .116 | .772 |
|  |  | PD ~ ID + Outcome | .15 |  |
|  | Interaction | PD ~ ID + Outcome + First Trial + Outcome* First Trial | .033 | .287 |
|  |  | PD ~ ID + Outcome + First Trial | .116 |  |

*Table S8.* Model comparisons for the False Belief trials of the Action Prediction block in 9-months-olds (*N* = 65) and 18-months-olds (*N* = 62), testing for potential order effects (First Trial Congruency). Abbreviations: ME = Main Effect, PD = averaged Pupil Dilation response, ID = subject intercept.

**Action Prediction**

**True Belief – First Trial Congruency**

|  | | Model 0 (1st row) and Model 1 (2nd row) |  | |
| --- | --- | --- | --- | --- |
|  |  |  | BF | BF_10_ |
| 9 months  (*N* = 59) | ME *Outcome* | PD ~ ID + Outcome + First Trial | .034 | .131 |
|  |  | PD ~ ID + First Trial | .258 |  |
|  | ME *First Trial* | PD ~ ID + Outcome + First Trial | .034 | .236 |
|  |  | PD ~ ID + Outcome | .143 |  |
|  | Interaction | PD ~ ID + Outcome + First Trial + Outcome* First Trial | .008 | .234 |
|  |  | PD ~ ID + Outcome + First Trial | .034 |  |
| 18 months  (*N* = 58) | ME *Outcome* | PD ~ ID + Outcome + First Trial | .078 | .286 |
|  |  | PD ~ ID + First Trial | .272 |  |
|  | ME *First Trial* | PD ~ ID + Outcome + First Trial | .078 | .292 |
|  |  | PD ~ ID + Outcome | .267 |  |
|  | Interaction | PD ~ ID + Outcome + First Trial + Outcome* First Trial | .017 | .212 |
|  |  | PD ~ ID + Outcome + First Trial | .078 |  |

*Table S9.* Model comparisons for the True Belief trials of the Action Prediction block in 9-months-olds (*N* = 59) and 18-months-olds (*N* = 58), testing for potential order effects (First Trial Congruency). Abbreviations: ME = Main Effect, PD = averaged Pupil Dilation response, ID = subject intercept.

**S4. Pupil Dilation Results at 50% of samples as inclusion criteria.**

**Object Memory**

*Nine-months-olds.* We computed Bayesian LMMs contrasting the two outcomes at each time point starting from 1000ms post onset. This showed moderate to strong evidence for greater pupil dilation for the reality-congruent than the reality-incongruent outcome after the object started appearing (BF_10_ repeatedly exceeded our predefined threshold ranging between 3 and 20.74 in the time window between 2550 – 2708 ms and 3908 ms – 4558 ms). Similarly, when averaging across the entire 5 second outcome time window, 9-month-olds’ pupil size was larger during the reality-congruent than the reality-incongruent outcome in False Belief trials (BF_10_ = 32.53; congruent: *N* = 42, *M* = .016, *SD* = .03; incongruent: *N* = 44, *M* = -.001, *SD* = .017). This indicates that 9-months-old infants were surprised to see the object revealed in its current location and expected it to appear where the agent believed it to be. In contrast, there was evidence for higher pupil dilation for the reality incongruent compared to congruent outcome in a very short time window between 1275 ms – 1391 ms (BF_10_ between 3 and 12.63; averaged across time window: BF_10_ = .24; congruent: *N* = 28, *M* = .011, *SD* = .017; incongruent: *N* = 35, *M* = .019, *SD* = .028). Thus, in line with the altercentric theory, 9-months-olds seemed to misremember the object in its first location when the agent held a false belief, but not when the agent had witnessed the transfer (True Belief).

*Eighteen-months-olds.* In contrast to the 9-month-olds, the 18-month-old infants showed moderate to extreme evidence for a greater pupil dilation for the reality-incongruent compared to the reality-congruent outcome in False Belief trials (BF_10_ repeatedly exceeded our predefined threshold, ranging between 3 and 219.96 in the time window from 1500 ms to 2241 ms after the object started appearing). This indicates that 18-months-old infants were surprised about outcomes in which the object reappeared from the empty (reality incongruent) location. Thus, 18-month-olds showed the opposite pattern of 9-month-old infants, showing surprise when the scene violated reality. When averaging the pupil dilation across the whole 5 second outcome phase, there was moderate evidence against a difference between outcomes (BF_10_ = .16, congruent: *N* = 39, *M* = .016, *SD* = .026; incongruent: *N* = 36, *M* = .017, *SD* = .018). As for the younger infants, there was moderate evidence against a difference in the True Belief control condition (dynamical analysis: BF_10_ between .20 and 5.10; average across outcome phase: BF_10_ = .30, congruent: *N* = 33, *M* = .024, *SD* = .022; incongruent: *N* = 22, *M* = .019, *SD* = .023). Thus, in contrast to 9-months-olds, 18-months-olds did not show any altercentric memory error, but remembered the object in its correct location.

**Action Prediction**

*Nine-months-olds.* In the Action Prediction block, 9-month-olds showed no systematic differences in pupil dilation depending on outcome in False Belief trials (dynamic analysis: BF_10_ = between .17 and 24.33; average across outcome phase: BF_10_ = .19, congruent: *N* = 33, *M* = .007, *SD* = .02; incongruent: *N* = 36, *M* = .007, *SD =* .028). In the True Belief control condition, in turn, infants showed higher pupil response to incongruent than congruent outcomes in a time window ranging from around 2216 ms to 2700 ms (BF_10_ between 3 and 132.73). This indicates that 9-months-olds were surprised to see the agent search in the empty location when she knew where the object was (True Belief). The average pupil dilation did not differ between congruent and incongruent outcomes (True Belief: BF_10_ = .48, congruent: *N* = 34, *M* = .007, *SD* = .027; incongruent: *N* = 24, *M* = .015, *SD* = .015).

*Eighteen-months-olds.* The 18-months-olds showed evidence against differential pupil dilation depending on the outcome both in the False Belief and the True Belief trials in the Action Prediction block (dynamic analysis: False Belief: BF_10_ between .16 and 3.72; True Belief: BF_10_ between .17 and 2.34; average across outcome phase: False Belief: BF_10_ = .18, congruent: *N* = 41, *M* = .011, *SD* = .024; incongruent: *N* = 39, *M* = .013, *SD =* .031; True Belief: BF_10_ = .21, congruent: *N* = 36, *M* = .011, *SD* = .025; incongruent: *N* = 41, *M* = .013, *SD* = .023). Thus, 18-months-olds’ showed no sign of correct action prediction, neither when the agent held a true nor a false belief

**S5. Robustness check**

To evaluate the robustness of our statistical findings, we applied a reduced multiverse approach. The multiverse approach addresses the uncertainty created by certain data management choices and probes for the robustness of parameter estimates or statistical results across different alternative preprocessing decisions (Steegen et al., 2016; Gelman & Loken, 2014). Thus, by specifying a set of plausible alternative decisions the same hypothesis is tested with varying preprocessing steps or statistical tests. Such an approach allows to evaluate the impact of preprocessing decisions and helps to specify the most robust pattern of results. The multiverse approach has been applied to infant pupillometry data, as illustrated in Calignano et al., 2024; and Sirois et al., 2023. Here, we focus on three specific decisions in the preprocessing procedure: 1) the filter applied to the raw pupil data, 2) the baseline correction, and 3) the inclusion criterion of a minimum amount of sample points. Please note that a full multiverse analysis is outside the scope of the present study.

1. **First degree: Pupil size filtering**

As a first step, we created an alternative dataset to the dataset in the main manuscript by applying a different filter to the raw pupil data. Thus, as a first step, we either applied a 1) percentile cut-off filter (option A, see manuscript for details) or 2) a 5-point moving average filter, (option B).

1. **Second degree: Baseline correction**

At the end of step 1, we have obtained two different datasets, which subsequently underwent three different baseline correction time windows. In step 2, we either computed a baseline correction using a baseline segment of the last 500 ms of the anticipation phase (16.5-17 sec) as reported in the main manuscript (step 2, option A); a baseline segment of 500 ms that was recorded directly preceding the analyzed pupil response in the outcome phase (17.7 – 18.2 sec; step 2, option B); or a baseline segment of the last 250 ms of the anticipation phase (i.e., 16.75-17 sec; step 2, option C). Thus, after this second forking path, we have obtained a total of 6 datasets (step 1 with 2 options and step 2 with 3 options).

1. **Third degree: Inclusion criterion**

As a last step, we varied the inclusion criterion, i.e., the number of data points, that an infant had to contribute in order to undergo our main analysis. We subset the obtained 6 datasets further by only including infants who have contributed at least 10% of data points (step 3, option A); at least 50% of data points (step 3, option B); or who have contributed 80% of data points (step 3, option C) within the outcome phase (18.2-23.2 s). Accordingly, this created a multiverse of 18 plausible datasets, resulting from the three forking paths (step 1 x step 2 x step 3).

**Results**

*Nine-months-olds*

Table S10 shows the baseline-corrected pupil dilation effects in the False Belief condition of the Object Memory block in the 9-months-olds after different preprocessing steps. Dynamic analyses revealed that our statistical results were characterized by a strong robustness to the three preprocessing choices. Sixteen of the resulting 18 linear models revealed BF_10_ (range: 3 - 3287) repeatedly exceeding the predefined threshold in a similar time window as reported in the main manuscript, see Table S10.

| 1. **Cut-off filter** | | | | | 1. **Moving average** | | | | |
| --- | --- | --- | --- | --- | --- | --- | --- | --- | --- |
| **Multiverse** | **baseline** | **Min %** | **max BF_10_** | **Time (ms)** | **Multiverse** | **baseline** | **Min %** | **max BF_10_** | **Time (ms)** |
| 1 | A | A. 10 | 44.14 | 2217-4508 | 10 | A | A. 10 | 484.91 | 2383-4050 |
| 2 | B |  | 6.18 | 2583-2675 | 11 | B |  | 31.48 | 2342-2683 |
| 3 | C |  | 65.97 | 2213-4508 | 12 | C |  | 3287 | 2192-4042 |
| 4 | A | B. 50 | 20.56 | 2550-4900 | 13 | A | B. 50 | 165.42 | 2158-4100 |
| 5 | B |  | 3.55 | 2650-2667 | 14 | B |  | 20.94 | 2342-2667 |
| 6 | C |  | 26.27 | 2225-4958 | 15 | C |  | 540.5 | 2142-4025 |
| 7 | A | C. 80 | 15 | 2341-4575 | 16 | A | C. 80 | 28.42 | 2208-4200 |
| 8 | B |  | 1.78 | - | 17 | B |  | 4.92 | 2450-2708 |
| 9 | C |  | 22.01 | 2308-4517 | 18 | C |  | 196.47 | 1950-4017 |

*Table S10.* Comparison of the multiverse analysis, contrasting the impact of different preprocessing decisions on the effect observed in the False Belief Object Memory trials in the 9-month-olds. As a first step, we contrasted a cut-off percentile filter (A) with a moving average filter (B) and computed three different baselines in step 2 (A: 500 ms prior outcome phase, B: 500 ms baseline within outcome phase, C: 250 ms prior outcome phase). In step 3, three different inclusion criteria were defined by including infants with a minimum of A: 10%, B: 50%, or C: 80% of sample points during the outcome phase. For each multiverse, the maximum BF_10_ and the approximate time window of the effect is shown.

*Eighteen-months-olds*

Similarly, for the reality congruency effect in the 18-months-olds’, 18 out of 18 datasets showed BF_10_ exceeding 3 (range 3-624.68) in a time window spanning roughly 1300 to 2200 ms, see Table S11. Thus, similar to the 9-months-olds, dynamic analyses suggested a strong robustness to the three preprocessing choices in the 18-months-olds.

| 1. **Cut-off filter** | | | | | 1. **Moving average** | | | | |
| --- | --- | --- | --- | --- | --- | --- | --- | --- | --- |
| **Multiverse** | **baseline** | **Min %** | **max BF_10_** | **Time (ms)** | **Multiverse** | **baseline** | **Min %** | **max BF_10_** | **Time (ms)** |
| 1 | A | A. 10 | 277.61 | 1508-2242 | 10 | A | A. 10 | 2035 | 1358-2250 |
| 2 | B |  | 21.04 | 1325-3092 | 11 | B |  | 174.45 | 1233- 2175 |
| 3 | C |  | 471.79 | 1508-2233 | 12 | C |  | 624.68 | 1308-2425 |
| 4 | A | B. 50 | 215.29 | 1558-2242 | 13 | A | B. 50 | 2223 | 1391-2275 |
| 5 | B |  | 11.72 | 1258-1716 | 14 | B |  | 178.62 | 1033-2175 |
| 6 | C |  | 147.8 | 1525-2233 | 15 | C |  | 1594 | 1392-2450 |
| 7 | A | C. 80 | 17.97 | 1683-2225 | 16 | A | C. 80 | 116.36 | 1683-2258 |
| 8 | B |  | 9.79 | 1317-2125 | 17 | B |  | 12.83 | 1317-2200 |
| 9 | C |  | 16.97 | 1667-2217 | 18 | C |  | 54.67 | 1667-2250 |

*Table S11*. Comparison of the multiverse analysis, contrasting the impact of different preprocessing decisions on the effect observed in the False Belief Object Memory trials in the 18-month-olds. As a first step, we contrasted a cut-off percentile filter (A) with a moving average filter (B) and computed three different baselines in step 2 (A: 500 ms prior outcome phase, B: 500 ms baseline within outcome phase, C: 250 ms prior outcome phase). In step 3, three different inclusion criteria were defined by including infants with a minimum of A: 10%, B: 50%, or C: 80% of sample points during the outcome phase. For each multiverse, the maximum BF_10_ and the approximate time window of the effect is shown.

**S6. Control analysis for sample points during change of location**

To control for potential attentional differences during the change of location, we analyzed the number of data points during this time window (starting from 8.6 to 11s) This 2.4s time window corresponds to a total of 289 sample points. As described in the main manuscript, we included infants, who contributed at least 50% of the sample points during this time window.

In the Object Memory block, there was no difference between the number of samples provided by the 9-months-olds during the change of location time window in the True compared to False Belief trials (BF_10_ = .54; True Belief: *N* = 85, *M* = 269.72, *SD* = 36.29; False Belief: *N* = 115, *M* = 276.13, *SD* = 24.47). Similarly, in the Action Prediction block, there was no difference between the number of samples provided by the 9-months-olds (BF_10_ = 2.15; True Belief: *N* = 82, *M* = 258.9, *SD* = 44.97; False Belief: *N* = 97, *M* = 273.63, *SD* = 30.51).

Similarly, for the 18-month-old infants, there was no difference observed in the Object Memory block (BF_10_ = 1.01; True Belief: *N* = 68, *M* = 262.26; *SD* = 38.43; False Belief: *N* = 106, *M* = 273.32; *SD* = 30.45). In contrast, in the Action Prediction Block, 18-month-old infants contributed fewer sample points in the True Belief trials (BF_10_ = 10.51; True Belief: *N* = 90, *M* = 262.62; *SD* = 43.98; False Belief: *N* = 99, *M* = 277.38; *SD* = 25.68). Thus, there was more data loss in the 18-month-olds during the True Belief trials, suggesting that infants were less attentive during the change of location when the agent was present compared to when she missed the change.

As a follow up, we compared the congruent and incongruent outcome when only including infants who had observed 100% of the change of location in the True Belief trials of the Action Prediction block. Results showed no differences between the congruent and incongruent outcome, confirming the results of the main analyses (BF_10_ = .19 – 2.01).

**S7. Flow chart of sample size**

*
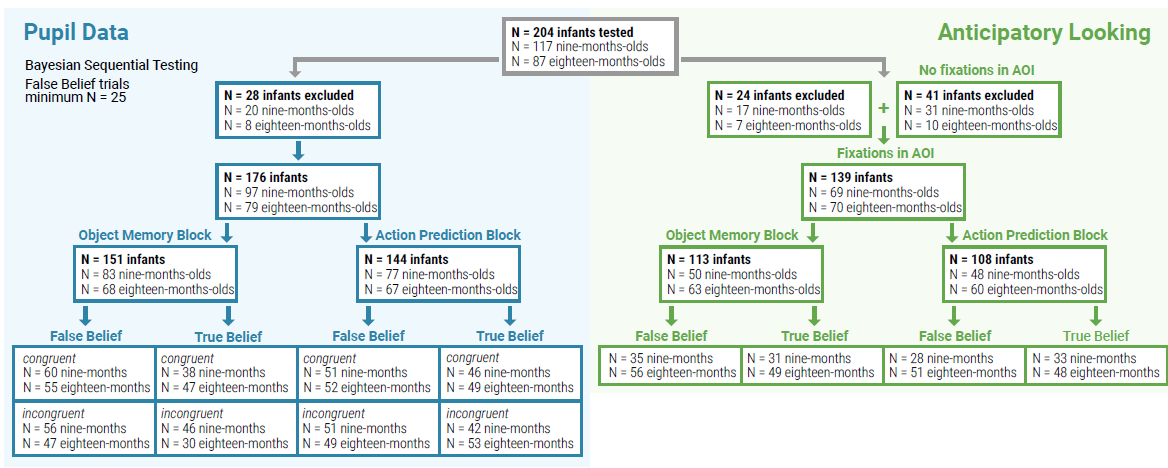
Figure S2*. Flow chart of participants tested and included in the present study across different analyses of pupil dilation and anticipatory looking.

**S8. Control analysis for missing data points**

After preprocessing, the complete dataset encompasses a total of 478800 datapoints, of which 146686 points were missing values (i.e., 30,6% of the time series). Figure S3 illustrates the trackloss across time separately for the two blocks and age groups in the different belief-outcome pairings. In 9-months-olds, an analysis of found sample points (in percentage) revealed no effect of Belief, or Outcome in neither the Object Memory (False Belief: BF_10_ = .29, congruent: *N* = 61, *M* = .69, *SD* = .27; incongruent: *N* = 60, *M* = .74, *SD* = .25; True Belief: BF_10_ = .252, congruent: *N* = 43, *M* = .70, *SD* = .274; incongruent: *N* = 47, *M* = .75, *SD* = .244), nor the Action Prediction block (False Belief: BF_10_ = .233, congruent: *N* = 51, *M* = .62, *SD* = .233; incongruent: *N* = 52, *M* = .67, *SD* = .243; True Belief: BF_10_ = .252, congruent: *N* = 46, *M* = .70, *SD* = .272; incongruent: *N* = 45, *M* = .62, *SD* = .316). For an illustration of missing data points distributions, see Fig. S4. Similarly, there was no effect of missing data points in 18-months-olds in the Object Memory Block (False Belief: BF_10_ = .126, congruent: *N* = 56, *M* = .72, *SD* = .27; incongruent: *N* = 52, *M* = .71, *SD* = .29; True Belief: BF_10_ = .206, *N* = 49, *M* = .69, *SD* = .277; incongruent: *N* = 30, *M* = .66, *SD* = .231), or the Action Prediction block (False Belief: BF_10_ = .146, congruent: *N* = 53, *M* = .70, *SD* = .256; incongruent: *N* = 49, *M* = .72, *SD* = .256; True Belief: BF_10_ = .266, *N* = 47, *M* = .75, *SD* = .221; incongruent: *N* = 53, *M* = .70, *SD*= .281).

*
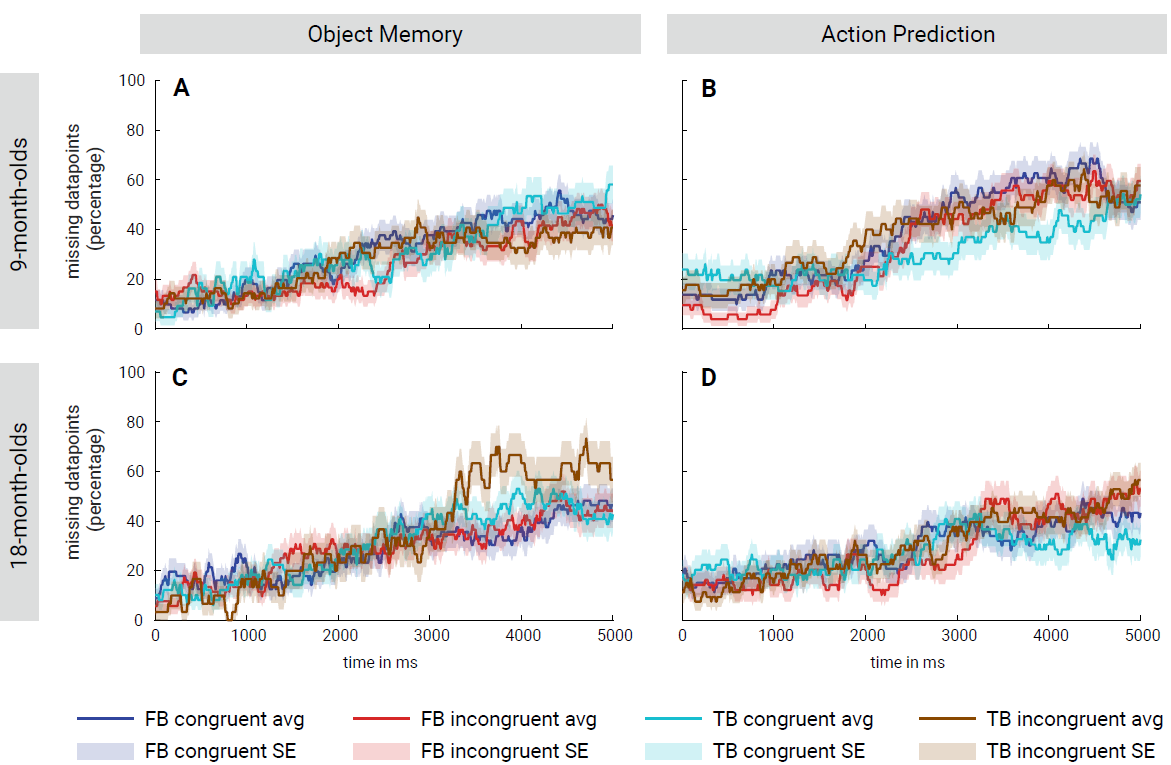
*

*Figure S3.* Number of missing data points in percentage by block (A, C) Object Memory, (B, D) Action Anticipation) and age group for the True and False Belief condition and reality congruent and incongruent outcomes across the outcome phase (5000 ms). Shadings represent the standard error (SE).


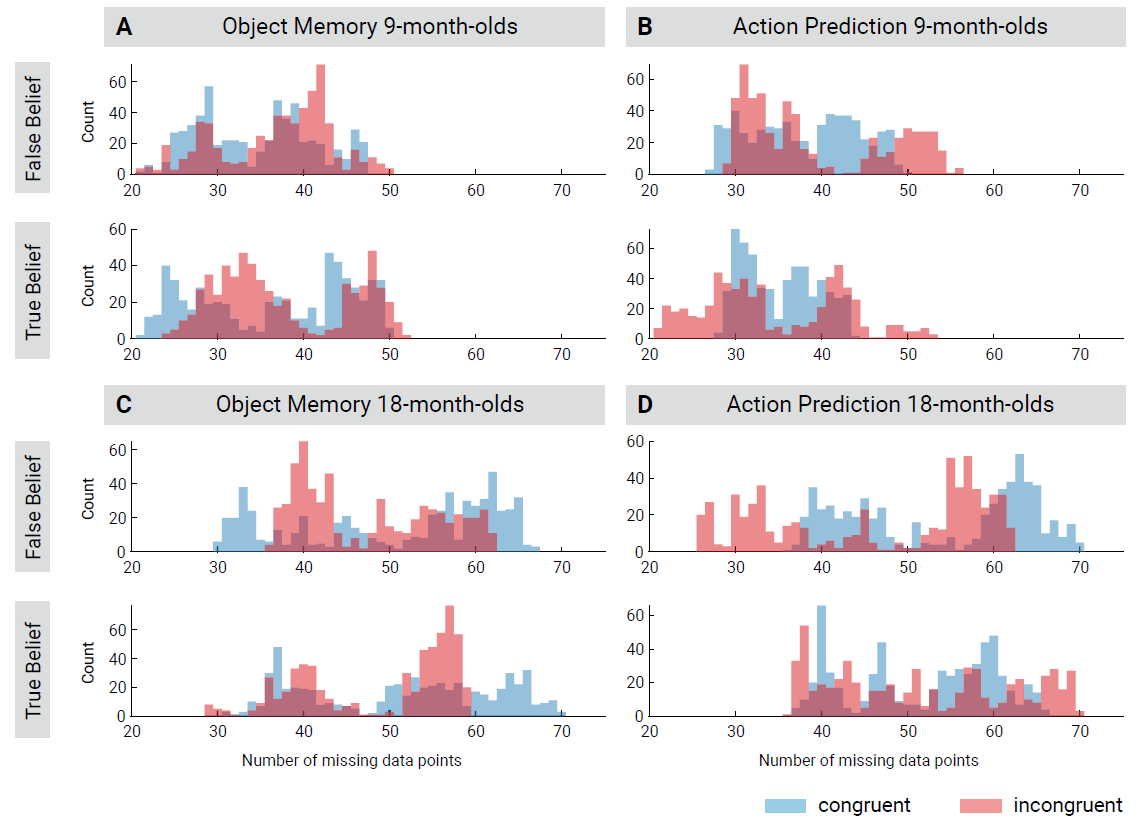
*Figure S4*. Distribution of the sum of missing data points by block (A) Object Memory, B) Action Anticipation) and age group (9-months olds: A, C; 18-months-olds: B, D) for the True and False Belief condition and reality congruent (blue) and incongruent (red) outcomes within the outcome phase.

**S9. Visualization of gaze points**

*
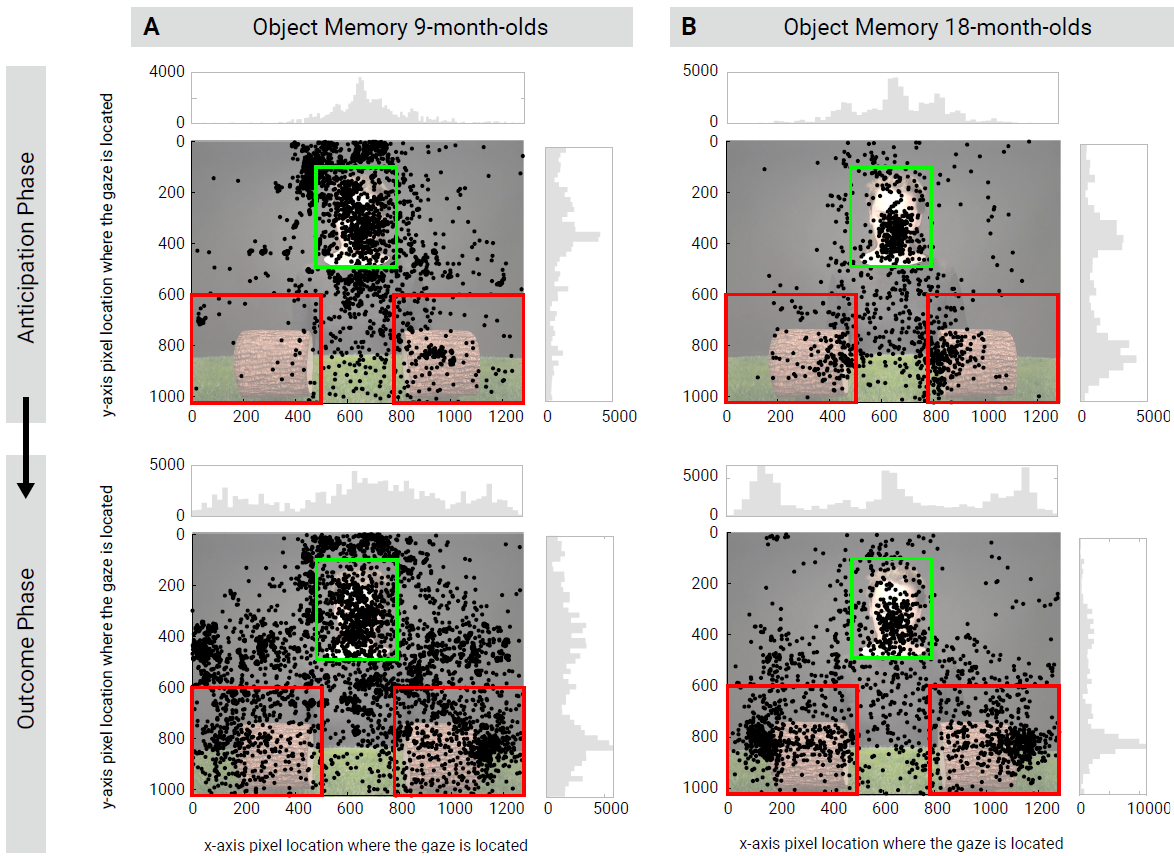
Figure S5*. Mapped fixation points corresponding to the pixel location on the X- and Y-axis of the pupil outcome time window (5000 ms) of the A) 9- and B) 18-months-olds included in the pupil analyses in the Object Memory block. The two red rectangles show the area of interested used for calculating the DLS in the anticipatory looking analysis (left or right location). The green rectangle shows the area of the agent’s face. Density plots are shown next to the X and Y axis in gray.

*
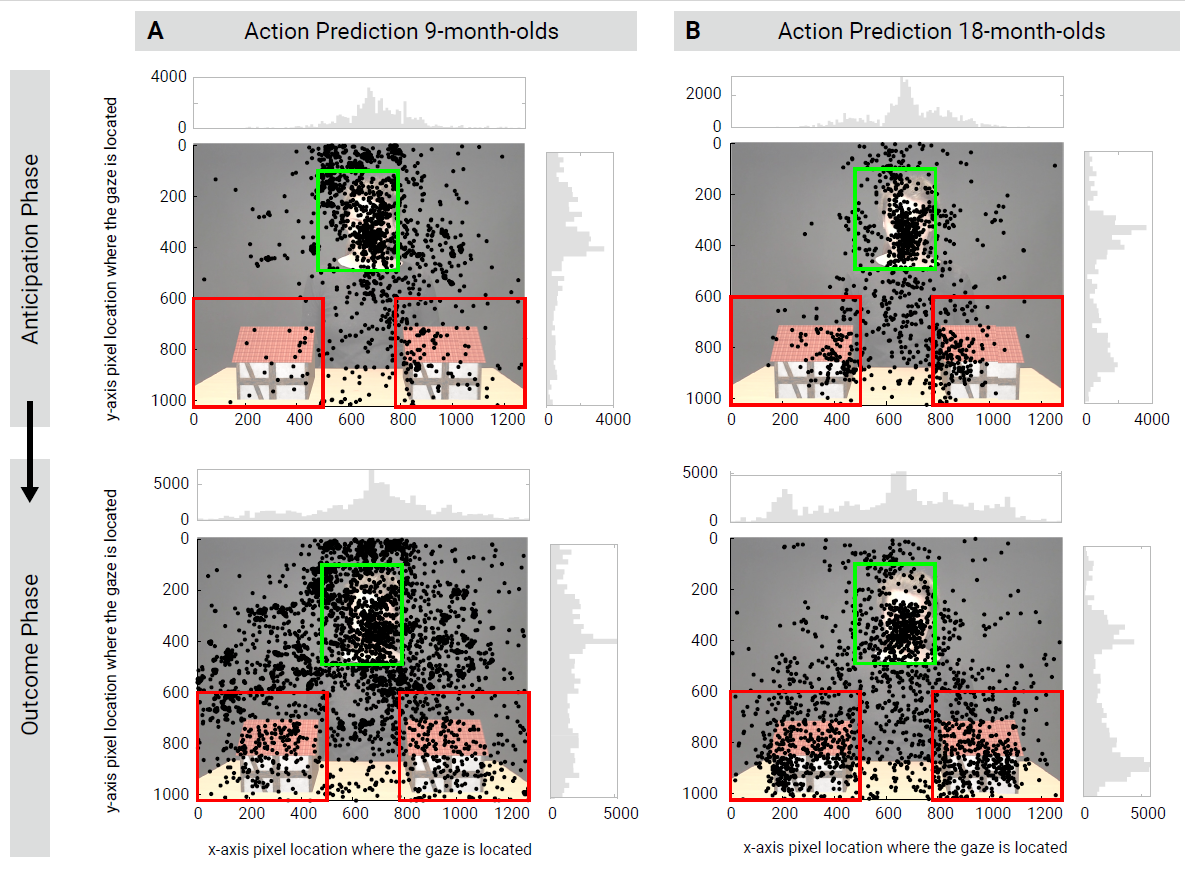
Figure S6*. Mapped fixation points corresponding to the pixel location on the X- and Y-axis of the pupil outcome time window (5000 ms) of the A) 9- and B) 18-months-olds included in the pupil analyses in the Action Prediction block. The two red rectangles show the area of interested used for calculating the DLS in the anticipatory looking analysis (left or right location). The green rectangle shows the area of the agent’s face. Density plots are shown next to the X and Y axis in gray.

**S10. QQ plots**

*
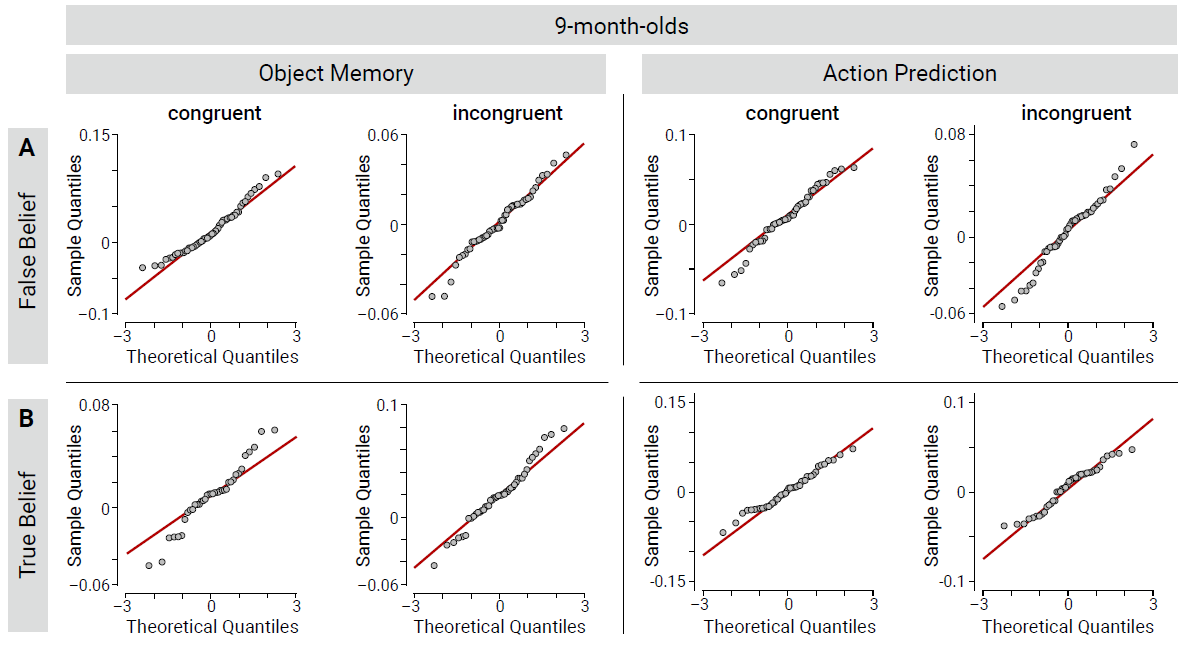
*

*Figure S*7. Distribution plots for the averaged pupil dilation response of the 9-months-olds for the A) False Belief and B) True Belief trials in the Object Memory and Action Prediction block, separated by Outcome (congruent, incongruent).

*
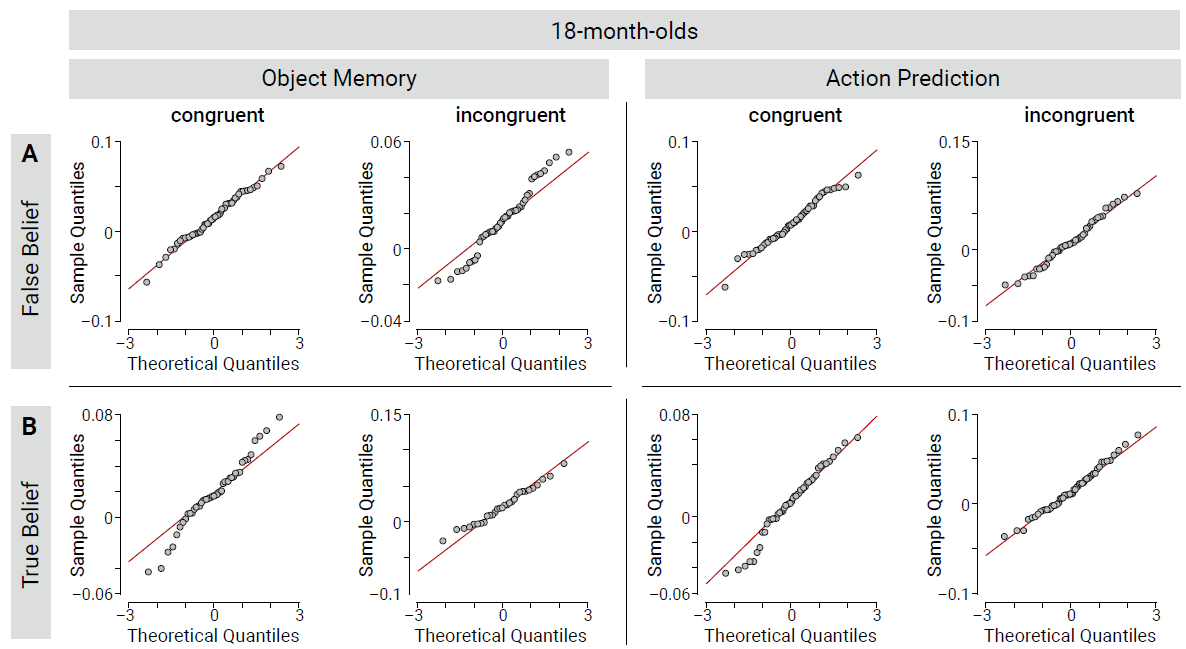
Figure S8*. Distribution plots for the averaged pupil dilation response of the 18-months-olds for the A) False Belief and B) True Belief trials in the Object Memory and Action Prediction block, separated by Outcome (congruent, incongruent).

**S11.Baseline-corrected pupil dilation averages**

|  |  |  |  | congruent | | |  | incongruent | | |
| --- | --- | --- | --- | --- | --- | --- | --- | --- | --- | --- |
|  |  |  | BF_10_ | *M* | *SD* | *N* |  | *M* | *SD* | *N* |
| 9 months | Object Memory | FB | 15.74 | .015 | .031 | 60 |  | .001 | .02 | 56 |
|  | Object Memory | TB | .33 | .009 | .024 | 38 |  | .019 | .026 | 46 |
|  | Action Prediction | FB | .27 | .009 | .03 | 51 |  | .004 | .026 | 51 |
|  | Action Prediction | TB | .15 | .003 | .031 | 46 |  | .005 | .024 | 42 |
| 18 months | Object Memory | FB | .14 | .015 | .026 | 55 |  | .016 | .018 | 47 |
|  | Object Memory | TB | .18 | .018 | .025 | 47 |  | .022 | .025 | 30 |
|  | Action Prediction | FB | .15 | .009 | .026 | 52 |  | .011 | .032 | 49 |
|  | Action Prediction | TB | .26 | .011 | .026 | 49 |  | .015 | .025 | 53 |

*Table S12*. Baseline-corrected pupil dilation average per age group and outcome scene (reality congruent vs incongruent) in the two blocks (Object Memory, Action Prediction) for the True and False belief trials. Results are shown along with their mean (*M),* standard deviation (*SD)* and underlying sample size (*N)*. The BF_10_ tests the contrast of congruent vs incongruent outcomes in False Belief (FB) trials and in True Belief (TB) trials.

**S12. Results for LMM Outcome x Belief**

As preregistered, we examined whether the pupil dilation was modulated by an interaction of infant’s expectations and the belief of the agent. To this end, we ran LMMs with the averaged pupil dilation (PD) as the dependent variable, *Outcome* and *Belief* as independent variables and a random intercept per subject for the two age groups separately.

**Object Memory**

*Nine-months-olds.* In the Object Memory block of the 9-months-olds, the LMM showed moderate evidence for an interaction of *Outcome* and *Belief* (BF_10_ = 4.54, see Table S13 for all BFs and model comparisons for the Object Memory block). Please note, however, that the BFs of the underlying Model 0 and Model 1 were small, thus the positive evidence for an interaction needs to be interpreted with caution. We found moderate evidence for a main effect of *Belief* (BF_10_ = 3.92) and against a main effect of *Outcome* (BF_10_ = .258).

*Eighteen-months-olds.* For the 18-months-olds, the LMM revealed moderate evidence against the main effects of *Outcome* and *Belief* (BF_10_ = .134 and BF_10_ = .20, respectively) and the interaction (BF_10_ = .161) in the Object Memory block, see Table S13.

**Action Prediction**

*Nine-months-olds*. The analyses of the Action Prediction block revealed moderate evidence against the main effects of *Outcome* and *Belief* (BF_10_ = .108 and BF_10_ = .117, respectively) and against an interaction of the two factors, BF_10_ = .251.

*Eighteen-months-olds*. Similarly, we found moderate evidence against the main effects of *Outcome* and *Belief* (BF_10_ = .153 and BF_10_ = .126, respectively) and the interaction (BF_10_ = .149) in the Action Prediction block (see Table S14 for all BFs and model comparisons). Thus, for the 18-months-olds, there were no systematic interaction effects on the pupillary responses between the Outcome and the Belief of the agent (True Belief, False Belief).

**Object Memory**

**Outcome x Belief**

|  | | Model 0 (1st row) and Model 1 (2nd row) |  | |
| --- | --- | --- | --- | --- |
|  |  |  | BF | BF_10_ |
| 9 months (*N* = 80) | ME *Outcome* | PD ~ ID + Outcome + Belief | .791 | .258 |
|  |  | PD ~ ID + Belief | 3.07 |  |
|  | ME *Belief* | PD ~ ID + Outcome + Belief | .791 | 3.92 |
|  |  | PD ~ ID + Outcome | .202 |  |
|  | Interaction | PD ~ ID + Outcome + Belief + Outcome*Belief | 3.59 | 4.54 |
|  |  | PD ~ ID + Outcome + Belief | .791 |  |
| 18 months (*N* = 66) | ME *Outcome* | PD ~ ID + Outcome + Belief | .024 | .134 |
|  |  | PD ~ ID + Belief | .178 |  |
|  | ME *Belief* | PD ~ ID + Outcome + Belief | .024 | .20 |
|  |  | PD ~ ID + Outcome | .119 |  |
|  | Interaction | PD ~ ID + Outcome + Belief + Outcome*Belief | .004 | .161 |
|  |  | PD ~ ID + Outcome + Belief | .024 |  |

*Table S13.* Table S13 shows the null model (Model 0) and the alternative model (Model 1) for different model comparisons with the respective Bayes Factors of the models (BF) and model comparisons (BF_10_) for the Object Memory block in 9-months-olds (*N* = 80) and 18-months-olds (*N* = 66). Abbreviations: ME = Main Effect, PD = averaged Pupil Dilation response, ID = subject intercept.

**Action Prediction**

**Outcome x Belief**

|  | | Model 0 (1st row) and Model 1 (2nd row) |  | |
| --- | --- | --- | --- | --- |
|  |  |  | BF | BF_10_ |
| 9 months  (*N* = 76) | ME *Outcome* | PD ~ ID + Outcome + Belief | .012 | .108 |
|  |  | PD ~ ID + Belief | .113 |  |
|  | ME *Belief* | PD ~ ID + Outcome + Belief | .012 | .117 |
|  |  | PD ~ ID + Outcome | .105 |  |
|  | Interaction | PD ~ ID + Outcome + Belief + Outcome*Belief | .003 | .251 |
|  |  | PD ~ ID + Outcome + Belief | .012 |  |
| 18 months  (*N* = 67) | ME *Outcome* | PD ~ ID + Outcome + Belief | .02 | .153 |
|  |  | PD ~ ID + Belief | .13 |  |
|  | ME *Belief* | PD ~ ID + Outcome + Belief | .02 | .126 |
|  |  | PD ~ ID + Outcome | .158 |  |
|  | Interaction | PD ~ ID + Outcome + Belief + Outcome*Belief | .003 | .149 |
|  |  | PD ~ ID + Outcome + Belief | .02 |  |

*Table S14.* Model comparisons for the Action Prediction block in 9-months-olds (*N* = 76) and 18-months-olds (*N* = 67), testing an interaction of Outcome and Belief. Abbreviations: ME = Main Effect, PD = averaged Pupil Dilation response, ID = subject intercept.

**S13. Anticipatory looking**

13.1. Areas of Interest for the DLS in the anticipation phase

13.2. Anticipatory Looking of 9-months-olds

Anticipatory looking behavior was quantified via the differential looking score (DLS) given by the cumulative looking time to the area of interest (AOI) of a reality congruent (correct)outcome (reappearance or action), minus the cumulative looking time to the AOI of a reality incongruent outcome (incorrect outcome), divided by the total cumulative looking time to both AOIs in the anticipation period (15-17s after onset). Thus, a score of +1 presents a maximum preference to location B (the object’s current location) whereas a score of -1 reveals maximum preference to location A (i.e., the empty location). Accordingly, a DLS of 0 presents no tendency to look to one AOI. AOIs were defined using equal-sized rectangles centered around locations A and B, encompassing the area from where the object reappeared to the border of the screen, with a size of 500×424 pixels, as illustrated in Fig S9. The average DLS of both available trials was calculated. In addition, we evaluated infants’ first gaze to track which of the two AOIs participants fixated first during the anticipation phase (see Table S16). The first gaze was coded as 1 if the infant first fixated the current object location), and 0 if their first fixation was on the empty location. If participants contributed data for both trials (reality congruent and incongruent outcomes) within a belief condition (True or False Belief), only the first trial of the respective belief condition was considered for analysis.

**S13.1. Areas of Interest for the DLS in the anticipation phase
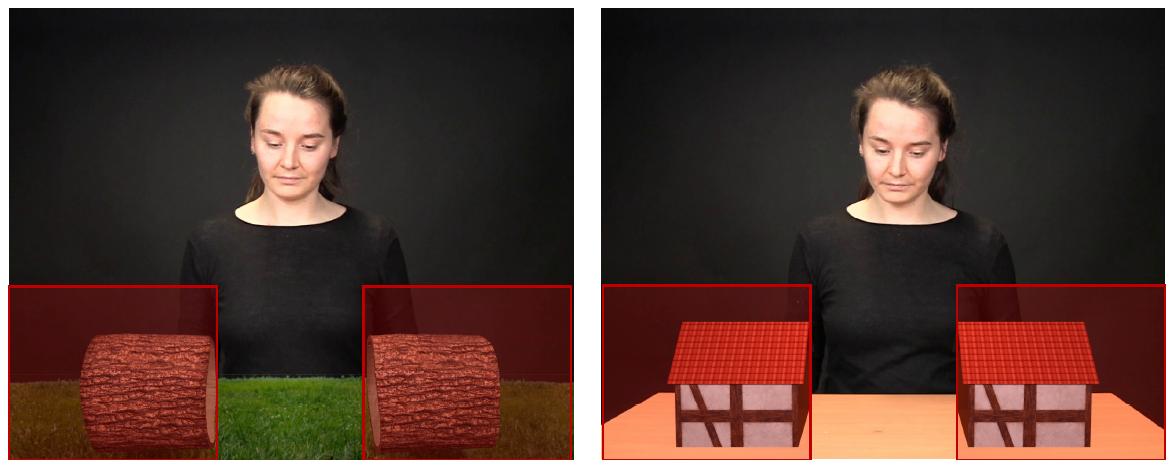
**

*Figure S9*. Figure S9 shows the two Areas of Interest (AOIs) for the DLS in the anticipation phase of the Object Memory (left) and Action Prediction block (right).

**S13.2. Anticipatory Looking**

*Nine-months-olds.* Nine-months-olds did not show any systematic anticipatory looking, potentially reflecting immature anticipatory looking behavior (Elsner & Adam, 2021; Krogh-Jespersen & Woodward, 2018; Reznick et al., 2000). The Wilcoxon Signed-Rank Test provided moderate evidence against preferential looking to the reality incongruent or congruent location in the younger age group in True Belief trials: BF_10_ = .09; *W* = 119; $\hat{R}$ = 1; *N* = 31; *M* = -.21; *SD* = .89; first gaze: BF_10_ = .19, see Table S15 and S16). The Bayes factor testing DLS>0 in False Belief trials was inconclusive, BF_10_ = .50; *W* = 281.5; $\hat{R}$ = 1; *N* = 35; *M* = .16; *SD* = .90; binomial test for first gaze: BF_10_ = .33; Similarly, we observed moderate evidence against preferential looking to the reality incongruent or congruent location in the younger age group in True Belief trials for the Action Prediction block (DLS>0 in True Belief trials: BF_10_ = .15; *W* = 174; $\hat{R}$ = 1; *N* = 33; *M* = -.09; *SD* = .91; first gaze: BF_10_ = .187). The Bayes factor testing DLS>0 in False Belief trials was inconclusive, BF_10_ = 1.31; *W* = 241.5; $\hat{R}$ = 1; *N* = 28; *M* = .34; *SD* = .86; binomial test for first gaze: BF_10_ = 1.32.

**Table: Differential looking score (DLS) in the anticipation phase**

|  |  | 9-months-olds | | | 18-months-olds | | |
| --- | --- | --- | --- | --- | --- | --- | --- |
|  |  | *N* | BF_10_ | *M (SD)* | *N* | BF_10_ | *M (SD)* |
| Object Memory | FB | 35 | .50 | .16 (.90) | 56 | 107.46 | .41 (.71) |
|  | TB | 31 | .09 | -.21 (.89) | 49 | 23.36 | .41 (.76) |
| Action Prediction | FB | 28 | 1.31 | .34 (.86) | 51 | 9.68 | .32 (.76) |
|  | TB | 33 | .16 | -.09 (.91) | 48 | 3.01 | .28 (.76) |

*Table S15*. Overview of the sample size *(N),* the BF_10_, the mean *(M)* and standard deviation *(SD)* of the averaged DLS for the two experimental blocks (Object Memory, Action Prediction) of the two age groups (9-months-olds, 18-month-olds) contrasting anticipatory looking behavior in False Belief (FB) and True Belief (TB) trials. The BF_10_ tests for DLS > 0.

**Table: First gaze in the anticipation phase**

|  |  | 9-months-olds | | | 18-months-olds | | |
| --- | --- | --- | --- | --- | --- | --- | --- |
|  |  | *N* | % | BF_10_ | *N* | % | BF_10_ |
| Object Memory | FB | 19/35 | 54.3 | .33 | 35/56 | 62.5 | 1.82 |
|  | TB | 15/31 | 48.4 | .19 | 37/49 | 75.5 | 244.02 |
| Action Prediction | FB | 18/28 | 64.3 | 1.32 | 32/51 | 62.7 | 1.72 |
|  | TB | 16/33 | 48.5 | .187 | 33/48 | 68.8 | 10.46 |

*Table S16*. Number and percentage of children that directed their first gaze towards the object’s (reality congruent) location in the anticipation phase, relative to the total *N*. Results are split per age group in the two blocks (Object Memory, Action Prediction) for the first False Belief (FB) and True Belief (TB) trial, respectively. The BF_10_ tests if the proportion is greater than chance level.

**S14. Results for LMM with Joint attention score**

To examine whether pupil dilation was associated with infants’ Joint Attention Score (JAS), we ran LMMs with the averaged pupil dilation (PD) as the dependent variable, *Outcome* and *JAS* as independent variables and a random intercept per subject.

**Object Memory**

*Nine-months-olds.* For the Object Memory block, the LMM showed very strong evidence against a main effect of *JAS* (False Belief: BF_10_ = .047; True Belief: BF_10_ = .06) and no interaction of *Outcome* and *JAS* (False Belief: BF_10_ = .126; True Belief: BF_10_ = .051), suggesting that infants’ JAS was not related to 9-month-olds’ pupil response. The BF_10_ for *Outcome* indicated moderate evidence against a main effect of *Outcome* in True Belief trials (BF_10_ = .198). The BF_10_ for False Belief trials was inconclusive (BF_10_ = 1.58, see Table S17 & S18 for all BFs and model comparisons for the Object Memory block).

*In Eighteen-months-olds.*, the BF testing for a main effect of JAS was inconclusive for the Object Memory False Belief trials (BF_10_ = .50). In True Belief trials, 18-months-olds showed moderate to extreme evidence against a main effect of *JAS* (BF_10_ = .034). The Bayes factor revealed moderate evidence against a main effect of *Outcome* (False Belief: BF_10_ = .291, True Belief: BF_10_ = .259), and against the interaction of *Outcome* and *JAS* in True Belief trials (BF_10_ = .09). The BF testing for an interaction of *JAS* and *Outcome* in False Belief trials was inconclusive, BF_10_ = .455, see Table S17 & S18 for all BFs and model comparisons for the Object Memory block.

**Object Memory**

**False Belief – Joint Attention Score**

|  | | Model 0 (1st row) and Model 1 (2nd row) |  | |
| --- | --- | --- | --- | --- |
|  |  |  | BF | BF_10_ |
| 9 months (*N* = 73) | ME *Outcome* | PD ~ ID + Outcome + JAS | .071 | 1.58 |
|  |  | PD ~ ID + JAS | .045 |  |
|  | ME *JAS* | PD ~ ID + Outcome + JAS | .071 | .047 |
|  |  | PD ~ ID + Outcome | 1.50 |  |
|  | Interaction | PD ~ ID + Outcome + JAS + Outcome* JAS | .009 | .126 |
|  |  | PD ~ ID + Outcome + JAS | .071 |  |
| 18 months (*N* = 62) | ME *Outcome* | PD ~ ID + Outcome + JAS | .121 | .291 |
|  |  | PD ~ ID + JAS | .417 |  |
|  | ME *JAS* | PD ~ ID + Outcome + JAS | .121 | .50 |
|  |  | PD ~ ID + Outcome | .264 |  |
|  | Interaction | PD ~ ID + Outcome + JAS + Outcome* JAS | .055 | .455 |
|  |  | PD ~ ID + Outcome + JAS | .121 |  |

*Table S17.* Table S17 shows the null model (Model 0) and the alternative model (Model 1) for different model comparisons with the respective Bayes Factors of the models (BF) and model comparisons (BF_10_) for the Object Memory False Belief trials in 9-months-olds (*N* = 73) and 18-months-olds (*N* = 62) and their association with infants’ *Joint Attention Score*. Abbreviations: ME = Main Effect, PD = averaged Pupil Dilation response, ID = subject intercept, JAS = Joint Attention Score.

**Object Memory**

**True Belief – Joint Attention Score**

|  | | Model 0 (1st row) and Model 1 (2nd row) |  | |
| --- | --- | --- | --- | --- |
|  |  |  | BF | BF_10_ |
| 9 months (*N* = 57) | ME *Outcome* | PD ~ ID + Outcome + JAS | .012 | .198 |
|  |  | PD ~ ID + JAS | .061 |  |
|  | ME *JAS* | PD ~ ID + Outcome + JAS | .012 | .06 |
|  |  | PD ~ ID + Outcome | .202 |  |
|  | Interaction | PD ~ ID + Outcome + JAS + Outcome* JAS | 6.08e-04 | .051 |
|  |  | PD ~ ID + Outcome + JAS | .012 |  |
| 18 months (*N* = 53) | ME *Outcome* | PD ~ ID + Outcome + JAS | .009 | .259 |
|  |  | PD ~ ID + JAS | .035 |  |
|  | ME *JAS* | PD ~ ID + Outcome + JAS | .009 | .034 |
|  |  | PD ~ ID + Outcome | .27 |  |
|  | Interaction | PD ~ ID + Outcome + JAS + Outcome* JAS | 8.13e-04 | .09 |
|  |  | PD ~ ID + Outcome + JAS | .009 |  |

*Table S18.* Model comparisons for the Object Memory True Belief trials in 9-months-olds (N = 57) and 18-months-olds (*N* = 53) and their association with infants’ *Joint Attention Score*. Abbreviations: ME = Main Effect, PD = averaged Pupil Dilation response, ID = subject intercept, JAS = Joint Attention Score.

**Action Prediction**

*Nine-months-olds.* For the Action Prediction block, the LMM for the 9-months-olds revealed moderate evidence against a main effect of *Outcome* (False Belief: BF_10_ = .20; True Belief: BF_10_ = .225, also see Table S19 & S20 for all BFs and model comparisons). Further, the LMM showed strong evidence against a main effect of *JAS* (False Belief: BF_10_ = .052; True Belief: BF_10_ = .034) and moderate evidence against an interaction of *Outcome* and *JAS* in False Belief (BF_10_ = .313). The BF testing for an interaction in True Belief trials was inconclusive, BF_10_ = .422. Accordingly, the JAS was unrelated to infants’ pupillary response in the Action Prediction block.

*Eighteen-months-olds.* For the Action Prediction block, the LMM revealed moderate evidence against a main effect of *Outcome* (False Belief: BF_10_ = .242; True Belief: BF_10_ = .192, also see Table S19 & S20 for all BFs and model comparisons). The JAS was unrelated to 18-month-olds’ pupil response in the Action Prediction block as indicated by moderate and very strong evidence against a main effect of *JAS* (False Belief: BF_10_ = .027; True Belief: BF_10_ = .151), and strong evidence against an interaction of *Outcome* and *JAS* (False Belief: BF_10_ = .054; True Belief: BF_10_ = .061).

**Action Prediction**

**False Belief – Joint Attention Score**

|  | | Model 0 (1st row) and Model 1 (2nd row) |  | |
| --- | --- | --- | --- | --- |
|  |  |  | BF | BF_10_ |
| 9 months (*N* = 65) | ME *Outcome* | PD ~ ID + Outcome + JAS | .01 | .20 |
|  |  | PD ~ ID + JAS | .05 |  |
|  | ME *JAS* | PD ~ ID + Outcome + JAS | .01 | .052 |
|  |  | PD ~ ID + Outcome | .194 |  |
|  | Interaction | PD ~ ID + Outcome + JAS + Outcome* JAS | .003 | .313 |
|  |  | PD ~ ID + Outcome + JAS | .01 |  |
| 18 months (*N* = 62) | ME *Outcome* | PD ~ ID + Outcome + JAS | .006 | .242 |
|  |  | PD ~ ID + JAS | .026 |  |
|  | ME *JAS* | PD ~ ID + Outcome + JAS | .006 | .027 |
|  |  | PD ~ ID + Outcome | .235 |  |
|  | Interaction | PD ~ ID + Outcome + JAS + Outcome* JAS | 3.443e-04 | .054 |
|  |  | PD ~ ID + Outcome + JAS | .006 |  |

*Table S19.* Model comparisons for the Action Prediction False Belief trials in 9-months-olds (*N* = 65) and 18-months-olds (*N* = 62) and their association with infants’ *Joint Attention Score* Abbreviations: ME = Main Effect, PD = averaged Pupil Dilation response, ID = subject intercept, JAS = Joint Attention Score.

**Action Prediction**

**True Belief – Joint Attention Score**

|  | | Model 0 (1st row) and Model 1 (2nd row) |  | |
| --- | --- | --- | --- | --- |
|  |  |  | BF | BF_10_ |
| 9 months (*N* = 59) | ME *Outcome* | PD ~ ID + Outcome + JAS | .007 | .225 |
|  |  | PD ~ ID + JAS | .032 |  |
|  | ME *JAS* | PD ~ ID + Outcome + JAS | .007 | .034 |
|  |  | PD ~ ID + Outcome | .21 |  |
|  | Interaction | PD ~ ID + Outcome + JAS + Outcome* JAS | .003 | .422 |
|  |  | PD ~ ID + Outcome + JAS | .007 |  |
| 18 months (*N* = 58) | ME *Outcome* | PD ~ ID + Outcome + JAS | .031 | .192 |
|  |  | PD ~ ID + JAS | .163 |  |
|  | ME *JAS* | PD ~ ID + Outcome + JAS | .031 | .151 |
|  |  | PD ~ ID + Outcome | .207 |  |
|  | Interaction | PD ~ ID + Outcome + JAS + Outcome* JAS | .002 | .061 |
|  |  | PD ~ ID + Outcome + JAS | .031 |  |

*Table S20.* Model comparisons for the Action Prediction True Belief trials in 9-months-olds (*N* = 59) and 18-months-olds (*N* = 58) and their association with infants’ *Joint Attention Score*. Abbreviations: ME = Main Effect, PD = averaged Pupil Dilation response, ID = subject intercept, JAS = Joint Attention Score.

**S15. Relation with Mirror Self-recognition (MSR)**

To examine if the 18-months-olds’ pupil dilation was associated with a developing self-concept as assessed by mirror-self-recognition, we compared the pupil dilation in reaction to congruent vs incongruent outcomes and conducted LMMs using the average pupil dilation (PD) as the dependent variable, *Outcome* and *Mirror Self-recognition (MSR)* as independent variables and a random intercept per subject. Note that these analyses have to be interpreted with caution as the sample size was very small (mirror-recognizer, *N* = 16; mirror non-recognizer, *N* = 10).

**Object Memory**

The analysis of Object Memory block showed moderate evidence against a main effect of *Outcome* in False Belief (BF_10_ = .221), and True Belief trials (BF_10_ = .311). There was no indication of a main effect of *MSR* (False Belief*:* BF_10_ = .085; True Belief: BF_10_ = .12), and moderate evidence against an interaction of the two factors in False Belief trials (BF_10_ = .133). For True Belief trials, the Bayes Factor was inconclusive (BF_10_ = 2.17, see Table S21).

**Action Prediction**

Similarly, the analysis of the Action Prediction block showed no indication of a main effect of *MSR* (False Belief*:* BF_10_ = .286; True Belief: BF_10_ = .088). The Bayes factor testing for a main effect of *Outcome* was inconclusive (False Belief: BF_10_ = .427; True Belief: BF_10_ = .433). There was moderate evidence against an interaction of the two factors in False Belief trials (BF_10_ = .156). For True Belief trials, the Bayes Factor was inconclusive (BF_10_ = .474, see Table S22).

**Object Memory – Mirror Self-recognition**

|  | | Model 0 (1st row) and Model 1 (2nd row) |  | |
| --- | --- | --- | --- | --- |
|  |  |  | BF | BF_10_ |
| FB (*N* = 21) | ME *Outcome* | PD ~ ID + Outcome + MSR | .018 | .221 |
|  |  | PD ~ ID + MSR | .08 |  |
|  | ME *MSR* | PD ~ ID + Outcome + MSR | .018 | .085 |
|  |  | PD ~ ID + Outcome | .207 |  |
|  | Interaction | PD ~ ID + Outcome + MSR + Outcome* MSR | .002 | .133 |
|  |  | PD ~ ID + Outcome + MSR | .018 |  |
| TB (*N* = 19) | ME *Outcome* | PD ~ ID + Outcome + MSR | .036 | .311 |
|  |  | PD ~ ID + MSR | .117 |  |
|  | ME *MSR* | PD ~ ID + Outcome + MSR | .036 | .12 |
|  |  | PD ~ ID + Outcome | .302 |  |
|  | Interaction | PD ~ ID + Outcome + MSR + Outcome*MSR | .079 | 2.17 |
|  |  | PD ~ ID + Outcome + MSR | .036 |  |

*Table S21.* Table S21 shows the null model (Model 0) and the alternative model (Model 1) for different model comparisons with the respective Bayes Factors of the models (BF) and model comparisons (BF_10_) for the Object Memory False and True Belief trials in 18-months-olds (FB: *N* = 21; TB: *N* = 19) and their potential association with infants’ *Mirror Self-recognition*. Abbreviations: ME = Main Effect, PD = averaged Pupil Dilation response, ID = subject intercept, MSR = Mirror Self-recognition.

**Action Prediction – Mirror Self-recognition**

|  | | Model 0 (1st row) and Model 1 (2nd row) |  | |
| --- | --- | --- | --- | --- |
|  |  |  | BF | BF_10_ |
| FB (*N* = 18) | ME *Outcome* | PD ~ ID + Outcome + MSR | .114 | .427 |
|  |  | PD ~ ID + MSR | .268 |  |
|  | ME *MSR* | PD ~ ID + Outcome + MSR | .114 | .286 |
|  |  | PD ~ ID + Outcome | .40 |  |
|  | Interaction | PD ~ ID + Outcome + MSR + Outcome* MSR | .018 | .156 |
|  |  | PD ~ ID + Outcome + MSR | .114 |  |
| TB (*N* = 17) | ME *Outcome* | PD ~ ID + Outcome + MSR | .037 | .433 |
|  |  | PD ~ ID + MSR | .086 |  |
|  | ME *MSR* | PD ~ ID + Outcome + MSR | .037 | .088 |
|  |  | PD ~ ID + Outcome | .424 |  |
|  | Interaction | PD ~ ID + Outcome + MSR + Outcome*MSR | .018 | .474 |
|  |  | PD ~ ID + Outcome + MSR | .037 |  |

*Table S22.* Model comparisons for the Action Prediction False and True Belief trials in 18-months-olds (FB: *N* = 18; TB: *N* = 17) and their association with *Mirror Self-Recognition*. Abbreviations: ME = Main Effect, PD = averaged Pupil Dilation response, ID = subject intercept, MSR = Mirror Self-recognition.

**S16. Relation with age**

To examine whether pupil dilation and the altercentric bias was modulated by age as a continuous variable, we ran LMMs with the averaged pupil dilation (PD) as the dependent variable, *Outcome* and *Age in days* as independent variables and a random intercept per subject.

For the Object Memory block, the LMM showed moderate evidence against a main effect of *Outcome* in the True Belief trials (BF_10_ = 301; see Table S23 for all BFs and model comparisons for the Object Memory block) and inconclusive evidence in the False Belief trials (BF_10_ = .355). There was extreme evidence against a main effect of *Age* (False Belief: BF_10_ = 1.20e-04; True Belief: BF_10_ = 8.97e-05) and against an interaction of *Outcome* and *Age* (False Belief: BF_10_ = 4.05e-04; True Belief: BF_10_ = 1.34e-04, see Table S23).

For the Action Prediction block, the LMM revealed moderate evidence against a main effect of *Outcome* in both False Belief (BF_10_ = .111) and True Belief trials (BF_10_ = .205, see Table S24 for all BFs and model comparisons for the Action Prediction block). Further, the LMM showed extreme evidence against a main effect of *Age* (False Belief: BF_10_ = 7.39e-05; True Belief: BF_10_ = 3.08e-04) and against an interaction of *Outcome* and *Age* (False Belief: BF_10_ = 1.23e-04; True Belief: BF_10_ = 6.45e-05, see Table S24).

**Object Memory**

**Age (in days)**

|  | | Model 0 (1st row) and Model 1 (2nd row) |  | |
| --- | --- | --- | --- | --- |
|  |  |  | BF | BF_10_ |
| FB  (*N* = 135) | ME *Outcome* | PD ~ ID + Outcome + Age | 4.27e-05 | .355 |
|  |  | PD ~ ID + Age | 1.20e-04 |  |
|  | ME *Age* | PD ~ ID + Outcome + Age | 4.27e-05 | 1.20e-04 |
|  |  | PD ~ ID + Outcome | .356 |  |
|  | Interaction | PD ~ ID + Outcome + Age + Outcome*Age | 1.73e-08 | 4.05e-04 |
|  |  | PD ~ ID + Outcome + Age | 4.27e-05 |  |
| TB  (*N* = 111) | ME *Outcome* | PD ~ ID + Outcome + Age | 2.60e-05 | .301 |
|  |  | PD ~ ID + Age | 8.64e-05 |  |
|  | ME *Age* | PD ~ ID + Outcome + Age | 2.60e-05 | 8.97e-05 |
|  |  | PD ~ ID + Outcome | .29 |  |
|  | Interaction | PD ~ ID + Outcome + Age + Outcome*Age | 3.47e-09 | 1.34e-04 |
|  |  | PD ~ ID + Outcome + Age | 2.60e-05 |  |

*Table S23.* Table S23 shows the null model (Model 0) and the alternative model (Model 1) for different model comparisons with the respective Bayes Factors of the models (BF) and model comparisons (BF_10_) for the Object Memory block in False Belief trials (*N* = 135) and True Belief trials (*N* = 111) and their potential association with age. Abbreviations: FB = False Belief, TB = True Belief, ME = Main Effect, PD = averaged Pupil Dilation response, ID = subject intercept.

**Action Prediction**

**Age (in days)**

|  | | Model 0 (1st row) and Model 1 (2nd row) |  | |
| --- | --- | --- | --- | --- |
|  |  |  | BF | BF_10_ |
| FB  (*N* = 127) | ME *Outcome* | PD ~ ID + Outcome + Age | 7.79e-06 | .111 |
|  |  | PD ~ ID + Age | 7.02e-05 |  |
|  | ME *Age* | PD ~ ID + Outcome + Age | 7.79e-06 | 7.39e-05 |
|  |  | PD ~ ID + Outcome | .105 |  |
|  | Interaction | PD ~ ID + Outcome + Age + Outcome*Age | 9.59e-10 | 1.23e-04 |
|  |  | PD ~ ID + Outcome + Age | 7.79e-06 |  |
| TB  (*N* = 118) | ME *Outcome* | PD ~ ID + Outcome + Age | 6.61e-05 | .205 |
|  |  | PD ~ ID + Age | 3.22e-04 |  |
|  | ME *Age* | PD ~ ID + Outcome + Age | 6.61e-05 | 3.08e-04 |
|  |  | PD ~ ID + Outcome | .214 |  |
|  | Interaction | PD ~ ID + Outcome + Age + Outcome*Age | 4.26e-09 | 6.45e-05 |
|  |  | PD ~ ID + Outcome + Age | 6.61e-05 |  |

*Table S24.* Model comparisons for the Action Prediction block in False Belief trials (*N* = 127) and True Belief trials (*N* = 118) and their association with age. Abbreviations: FB = False Belief, TB = True Belief, ME = Main Effect, PD = averaged Pupil Dilation response, ID = subject intercept.

**S17. Within subject analysis**

To examine whether there was an altercentric bias in a within-subject-analysis, we ran pairwise Bayesian t-tests for each time point, contrasting the effect of *Outcome* for the Belief conditions and the two age groups separately. These yielded qualitatively similar results to the between-subject analyses reported in the Results section.

**Object Memory**

*Nine-months-olds.* There was moderate to strong evidence for greater pupil dilation for the reality-congruent than the reality-incongruent outcome (BF_10_ repeatedly exceeded our predefined threshold ranging between 3 and 10.16 in the time window 3842 ms – 4550 ms). When averaging across the entire 5 second time window, 9-month-olds’ pupil size was larger during the reality-congruent than the reality-incongruent outcome in False Belief trials (BF_10_ = 3.35; congruent: *N* = 43, *M* = .012, *SD* = .03; incongruent: *M* = .001, *SD* = .02). As predicted by the altercentric theory, this indicates that 9-months-old infants were surprised to see the object revealed in its current location and expected it to appear where the agent believed it to be. In contrast, there was evidence against such a difference in pupil responses between outcomes in the True Belief trials (dynamic analyses: BF_10_ between .08 and 2.76; average across outcome phase: BF_10_ = .13, congruent: *N* = 27, *M* = .013, *SD* = .022; incongruent: *M* = .01, *SD* = .023).

*Eighteen-months-olds.* In contrast to the 9-month-olds, the 18-month-old infants showed moderate to very strong evidence for a greater pupil dilation for the reality-incongruent compared to the reality-congruent outcome in False Belief trials (BF_10_ repeatedly exceeded our predefined threshold, ranging between 3 and 79.26 in the time window from 1350 ms to 2241 ms after the object started appearing). This indicates that 18-months-old infants were surprised about outcomes in which the object reappeared from the empty (reality incongruent) location. Thus, 18-month-olds showed the opposite pattern of 9-month-old infants, showing surprise when the scene violated reality. Again, we computed the average pupil dilation across the whole 5 second time window after object appearance, resulting in moderate evidence against a difference between outcomes for False Belief trials (BF_10_ = .13, congruent: *N* = 40, *M* = .014, *SD* = .025; incongruent: *M* = .016, *SD* = .018). As for the younger infants, there was moderate evidence against a difference in the True Belief control condition (dynamical analysis: BF_10_ between .07 and 2.09; average across outcome phase: BF_10_ = .16, congruent: *N* = 24, *M* = .021, *SD* = .025; incongruent: *M* = .019, *SD* = .022).

**Action Prediction**

*Nine-months-olds.* The Bayesian t-test comparing the averaged pupil dilation to congruent and incongruent outcomes in False Belief trials within subjects was inconclusive (dynamic analyses: BF_10_ between .12 and 8.20; average across outcome phase: BF_10_ = .43, congruent: *N* = 37, *M* = .008, *SD* = .028; incongruent: *M* = .004, *SD =* .028). In contrast, the 9-months-olds showed moderate to very strong evidence for a greater pupil dilation for the reality-incongruent compared to the reality-congruent outcome in True Belief trials (BF_10_ ranging between 3 and 58.36 in the time window from 2225 ms to 2742 ms, average: BF_10_ = .17, congruent: *N* = 29, *M* = .007, *SD* = .026; incongruent: *M* = .005, *SD* = .024).

*Eighteen-months-olds.* The 18-months-olds showed evidence against differential pupil dilation depending on the outcome in False Belief trials, i.e., 18-months-olds’ pupil size increased similarly for both the congruent and the incongruent outcome in the *Action* *Prediction* block (dynamic analyses: BF_10_ ranging between .06 and 1.83; average across outcome phase: BF_10_ = .12, congruent: *N* = 39, *M* = .009, *SD* = .022; incongruent: *M* = .011, *SD =* .028). Bayesian t-test comparing the pupil response in True Belief trials was inconclusive (dynamic analyses: BF_10_ ranging between .06 and 3.77, average across outcome phase: BF_10_ = .63, congruent: *N* = 44, *M* = .01, *SD* = .026; incongruent: *M* = .015, *SD =* .026).

**S18. Association between Object Memory and Action Prediction**

To investigate the relation between the observed altercentric bias in Object Memory and infants’ action prediction in False Belief trials and test whether the bias would predict infants’ correct action anticipation, we computed Bayesian correlations. The Bayes Factor testing for a relation between infants’ pupil dilation in response to reality congruent outcomes in the False Belief trials in the Object Memory and Action Prediction block was inconclusive in the younger age group (BF_10_ = .57, *r* = -.31, *N* = 31), and there was moderate evidence against a correlation in the older age group (BF_10_ = .23, *r* = .18, *N* = 40). Accordingly, the pupil response of 18-months-olds in the Object Memory False Belief trial was not predictive of their pupil dilation in the Action Prediction False Belief trial. The Bayes Factor testing for a relation between infants’ pupil dilation in the Object Memory block (difference in pupil dilation to reality congruent and incongruent outcomes) and their anticipatory looking in the False Belief trial of the Action Prediction block revealed moderate evidence against a correlation in the younger age group (BF_10_ = .30, *r* = -.25, *N* = 47), and older age group (BF_10_ = .15, *r* = .08, *N* = 45). Infants’ anticipatory looking in False Belief trials was not related between the Object Memory and Action Prediction block (younger age group: BF_10_ = .46, *r* = .10, *N* = 12; older age group: BF_10_ = .39, *r* = .12, *N* = 41).
